# Thiophenecarboxamide Derivatives Activated by EthA Kill *Mycobacterium tuberculosis* by Inhibiting the CTP Synthetase PyrG

**DOI:** 10.1016/j.chembiol.2015.05.016

**Published:** 2015-07-23

**Authors:** Giorgia Mori, Laurent R. Chiarelli, Marta Esposito, Vadim Makarov, Marco Bellinzoni, Ruben C. Hartkoorn, Giulia Degiacomi, Francesca Boldrin, Sean Ekins, Ana Luisa de Jesus Lopes Ribeiro, Leonardo B. Marino, Ivana Centárová, Zuzana Svetlíková, Jaroslav Blaško, Elena Kazakova, Alexander Lepioshkin, Nathalie Barilone, Giuseppe Zanoni, Alessio Porta, Marco Fondi, Renato Fani, Alain R. Baulard, Katarína Mikušová, Pedro M. Alzari, Riccardo Manganelli, Luiz Pedro S. de Carvalho, Giovanna Riccardi, Stewart T. Cole, Maria Rosalia Pasca

**Affiliations:** 1 Department of Biology and Biotechnology “Lazzaro Spallanzani”, University of Pavia, 27100 Pavia, Italy; 2 A. N. Bakh Institute of Biochemistry, Russian Academy of Science, 119071 Moscow, Russia; 3 Institut Pasteur, Unité de Microbiologie Structurale, CNRS-UMR3528, Université Paris Diderot, Sorbonne Paris Cité, 25 rue du Dr. Roux, 75724 Paris Cedex 15, France; 4 Global Health Institute, Ecole Polytechnique Fédérale de Lausanne, Station 19, 1015 Lausanne, Switzerland; 5 Department of Molecular Medicine, University of Padova, 35128 Padua, Italy; 6 Collaborative Drug Discovery, 1633 Bayshore Highway, Suite 342, Burlingame, CA 94010, USA; 7 Francis Crick Institute, Mill Hill Laboratory, The Ridgeway, Mill Hill, London NW7 1AA, UK; 8 Faculty of Pharmaceutical Sciences, UNESP - Univ Estadual Paulista, Araraquara, São Paulo 14801-902, Brazil; 9 Department of Biochemistry, Faculty of Natural Sciences, Comenius University in Bratislava, Ilkovičova 6, Mlynská dolina, 84215 Bratislava, Slovakia; 10 Institute of Chemistry, Faculty of Natural Sciences, Comenius University in Bratislava, Ilkovičova 6, Mlynská dolina, 84215 Bratislava, Slovak Republic; 11 Department of Chemistry, University of Pavia, 27100 Pavia, Italy; 12 Department of Biology, University of Florence, Sesto Fiorentino, Florence 50019, Italy; 13 Institut Pasteur de Lille, Center for Infection and Immunity, 59019 Lille, France

## Abstract

To combat the emergence of drug-resistant strains of *Mycobacterium tuberculosis*, new antitubercular agents and novel drug targets are needed. Phenotypic screening of a library of 594 hit compounds uncovered two leads that were active against *M*. *tuberculosis* in its replicating, non-replicating, and intracellular states: compounds 7947882 (5-methyl-*N*-(4-nitrophenyl)thiophene-2-carboxamide) and 7904688 (3-phenyl-*N*-[(4-piperidin-1-ylphenyl)carbamothioyl]propanamide). Mutants resistant to both compounds harbored mutations in *ethA* (*rv3854c*), the gene encoding the monooxygenase EthA, and/or in *pyrG* (*rv1699*) coding for the CTP synthetase, PyrG. Biochemical investigations demonstrated that EthA is responsible for the activation of the compounds, and by mass spectrometry we identified the active metabolite of 7947882, which directly inhibits PyrG activity. Metabolomic studies revealed that pharmacological inhibition of PyrG strongly perturbs DNA and RNA biosynthesis, and other metabolic processes requiring nucleotides. Finally, the crystal structure of PyrG was solved, paving the way for rational drug design with this newly validated drug target.

## Highlights

• Two compounds activated by EthA kill *M. tuberculosis* through PyrG inhibition
• EthA metabolite is active against PyrG and *M. tuberculosis* growth
• Definition of the mechanism of activation and validation of PyrG as a new drug target

## Graphical Abstract

**Figure:**
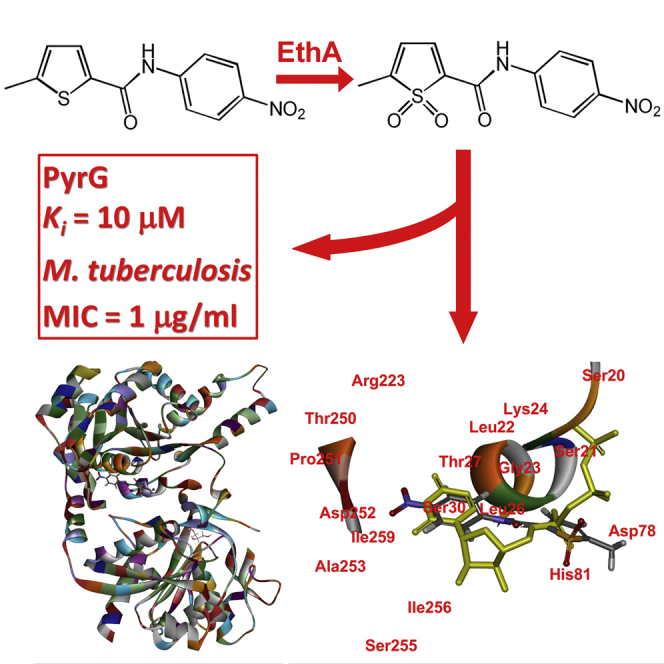

## Teaser

CTP synthetase PyrG, essential in *Mycobacterium tuberculosis*, could represent a new potential drug target. With a multidisciplinary approach, Mori et al. identify two compounds killing growing and dormant mycobacteria through PyrG inhibition, and define their mechanism of action.

## Introduction

Tuberculosis (TB) remains a leading cause of infectious mortality worldwide, killing approximately 1.5 million people each year. Drug-resistant strains of *Mycobacterium tuberculosis* threaten global TB management, with an estimated 450,000 cases being multidrug resistant, defined as resistant to rifampin and isoniazid. A subset of these cases, approximately 10%, is also resistant to the second-line drug classes, fluoroquinolones, and injectable aminoglycosides, and is referred to as extensively drug resistant (WHO, 2014).

Defining the pharmacological target(s) of antitubercular drugs under development and finding new compounds with greater potency are both important aspects in the search for agents that are effective against drug-sensitive and drug-resistant *M. tuberculosis* strains (Lechartier et al., 2014). Several current antimycobacterial agents are prodrugs requiring some form of cellular activation before they can bind to their specific targets and, in such cases, resistance can be mediated by mutations that prevent the activation step. Therefore, understanding the mode of activation not only helps to decipher the mechanisms of drug resistance, but may also facilitate the development of analogs that do not require activation (Dover et al., 2007).

In this work, by screening a library of compounds with known antitubercular activity, established by the National Institute of Allergy and Infectious Diseases (NIAID) (Ananthan et al., 2009; Goldman and Laughon, 2009; Maddry et al., 2009), a new series of molecules was found, displaying a very low minimum inhibitory concentration (MIC) value (0.5 μg/ml), that includes compounds 7947882 and 7904688. Through the isolation of *M. tuberculosis*-resistant mutants, genetic validation, and biochemical and structural studies, the main mechanisms of activation and resistance of these new antitubercular compounds have been characterized. The combined data indicate that 7947882 and 7904688 are prodrugs activated by the EthA monooxygenase, which then target PyrG, a cytidine triphosphate (CTP) synthetase catalyzing the ATP-dependent amination of uridine triphosphate (UTP) to form the essential pyrimidine nucleotide CTP (Long and Pardee, 1967). CTP synthetase is thus a tractable new TB drug target.

## Results and Discussion

### Screening of NIAID Library

A library of 594 compounds, selected by high-throughput screening (HTS) against *M. tuberculosis* H37Rv (Ananthan et al., 2009; Goldman and Laughon, 2009; Maddry et al., 2009), was tested for activity against non-replicating *M. tuberculosis* using the streptomycin-starved 18b (ss18b) model (Sala et al., 2010; Zhang et al., 2012). Two promising compounds were identified: a 5-methyl-*N*-(4-nitrophenyl)thiophene-2-carboxamide (7947882) and a 3-phenyl-*N*-[(4-piperidin-1-ylphenyl)carbamothioyl]propanamide (7904688). Both compounds also showed activity against replicating and intracellular *M. tuberculosis* H37Rv (Table 1). Moreover, the molecules were not cytotoxic to HepG2, A549, Raw, and Huh7 cell lines at concentrations below 40 μg/ml. Compounds were re-purchased from Chembridge Chemical Store (http://www.hit2lead.com/) and the results were confirmed.

### Isolation and Characterization of *M. tuberculosis*-Resistant Mutants

To characterize the mechanism of action of 7947882 and 7904688, several spontaneous *M. tuberculosis* mutants resistant to the compounds were isolated. The spontaneous mutants exhibited the same resistance levels to both drugs (10 μg/ml, 20× MIC) (Table 2). Illumina whole-genome sequencing of all mutants revealed mutations either in *ethA* (*rv3854c*), encoding a monooxygenase responsible for ethionamide (ETH) activation (Baulard et al., 2000), and/or *pyrG* (*rv1699*), encoding the CTP synthetase, which performs the ATP-dependent amination of UTP to form CTP as the final step of the pyrimidine nucleotide biosynthetic pathway (Endrizzi et al., 2004) (Table 2). Notably, *M. tuberculosis* mutants resistant to compound 7947882 carried different point mutations in *ethA*, resulting in either an amino acid substitution or a truncated protein. In addition, these mutants all harbored the same mutation in the *pyrG* gene: T557G (Val186Gly). By contrast, no mutations in *ethA* were found in *M. tuberculosis* mutants resistant to compound 7904688, but these all carried the Val186Gly substitution in PyrG (Table 2).

Since *pyrG*, unlike *ethA*, is predicted to be an essential gene in *M. tuberculosis* (Sassetti et al., 2001), it was hypothesized that EthA could be required to activate 7947882 and 7904688 compounds, while the target of the activated metabolites might be PyrG. The finding that all strains harboring a mutation in *ethA* showed cross-resistance to ETH, whereas strains mutated only in *pyrG* remained ETH sensitive, reinforced this hypothesis (Table 2).

### EthA Is an Activator of 7947882 and 7904688 Compounds

To verify whether EthA is responsible for the activation of 7947882 and 7904688, the *ethA* gene was cloned in the expression vector pSODIT-2, and *M. tuberculosis* H37Rv cells were transformed with the corresponding recombinant plasmid. A statistically significant shift in the MIC of the transformants was observed with respect to the control; overexpression of *ethA* in *M. tuberculosis* H37Rv increased the sensitivity to 7947882 and 7904688 (Table S1). Moreover, the overexpression of wild-type *ethA* restored the sensitivity to 7947882 in *M. tuberculosis* 82.14 mutant cells, carrying a mutation in *ethA* (Table S1).

To prove that both compounds were activated by EthA, a recombinant form of the *M. tuberculosis* enzyme was expressed in *Escherichia coli* and purified, and its activity toward the two compounds as substrates was assayed. EthA was active toward both 7947882 and 7904688, with *k*_cat_ values of 2.9 ± 0.08 and 2.4 ± 0.15 min^−1^ and *K*_m_ values of 0.037 ± 0.002 and 0.055 ± 0.004 mM for 7947882 and 7904688, respectively. Moreover, both compounds were better substrates for EthA than ETH, showing ∼10-fold higher affinity (*K*_m_ for ETH 0.34 mM), similar to that for phenylacetone, the best EthA substrate found so far (*K*_m_ 0.06 mM and *k*_cat_ 0.027 s^−1^) (Fraaije et al., 2004). The body of genetic and biochemical data strongly suggests that these two compounds are prodrugs that need EthA activation.

### 7947882 and 7904688 Do Not Affect PyrG Enzyme Activity but Require EthA Activation

To check whether compounds 7947882 and 7904688 were able to inhibit PyrG, their effect on the enzyme activity was evaluated. For this purpose, wild-type PyrG and the V186G mutant protein were produced in *E. coli*, purified, and characterized. *M. tuberculosis* PyrG shows catalytic constants (*k*_cat_ 21.9 ± 0.5 s^−1^ and *K*_m_ 0.18 ± 0.01 mM toward ATP; *k*_cat_ 22.9 ± 0.9 s^−1^ and *K*_m_ 0.14 ± 0.01 mM toward UTP) very similar to those of other bacterial CTP synthetases (Anderson, 1983; Long and Pardee, 1967; Willemoës et al., 2005). The PyrG mutant V186G was still active, but partially impaired, displaying reduced *k*_cat_ values toward both substrates (1.5 ± 0.11 and 1.6 ± 0.08 s^−1^ for ATP and UTP, respectively). Moreover, the mutant enzyme showed a *K*_m_ value for ATP that was about 10-fold higher than that of the wild-type protein (1.46 ± 0.18 mM), whereas the affinity for UTP was unchanged.

Since this mutation is associated with resistance to 7947882 and 7904688 (Table 2), it was conceivable that the ATP-binding site was involved in binding the inhibitors. For this reason, the effects of the two compounds were tested on wild-type PyrG at a final concentration of 200 μM. As expected for molecules that need to be activated by EthA, the compounds were ineffective toward PyrG in all the conditions tested.

**Figure 1:**
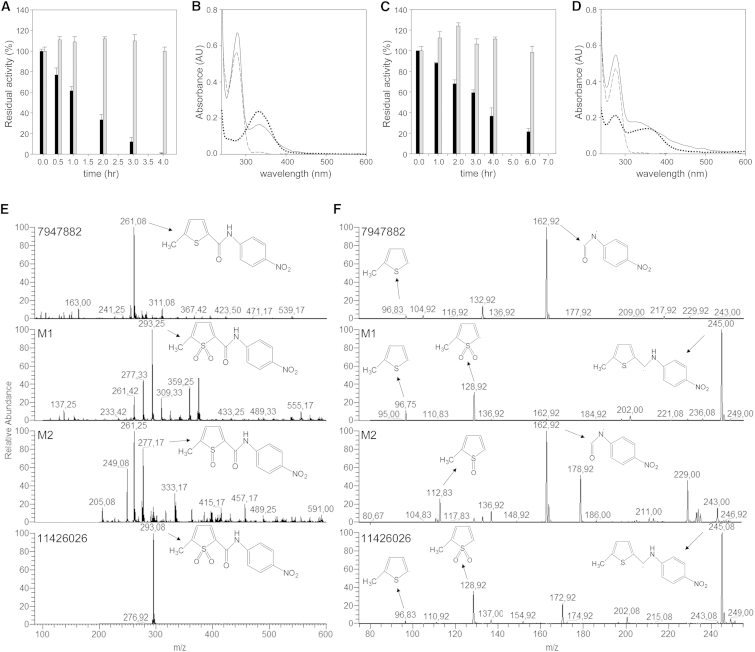
EthA Converts the 7947882 and 7904688 Compounds into Active PyrG Inhibitors (A) Inhibition of PyrG activity during the co-incubation with EthA and 7947882. Gray bars correspond to the activities of the blank controls in the absence of NADPH, and black bars represent the residual activities after incubation with working EthA. (B) UV-Vis spectra of the re-purified PyrG after co-incubation with EthA reaction with 7947882 compound. Solid line is the spectrum of PyrG incubated with full EthA reaction; dashed line is the spectrum of PyrG from blank reaction; dotted line is the spectrum of the compound at 20 μM. (C and D) Co-incubation of PyrG with EthA and 7904688 compound. Conditions are the same as for (A) and (B), respectively. (E and F) Identification of in vitro EthA metabolites of 7947882 compound. Mass spectrometry analysis (from top to bottom) of the 7947882 compound, the partially purified products of EthA reaction M1 and M2, and the synthetic metabolite 11426026. (E) Full electrospray ionization mass spectrometry of the compounds recorded in negative mode. (F) Fragmentation pattern of the compounds. See also Figures S1 and S2; Table S2.

Thus, to confirm that EthA produces metabolites that might act on PyrG, the EthA enzymatic reaction was performed with either 7947882 or 7904688 in the presence of PyrG, and the activity of the latter enzyme was monitored during the course of the reaction. The blank control was performed omitting reduced nicotinamide adenine dinucleotide phosphate (NADPH) to hinder the EthA-catalyzed reaction, and under these conditions PyrG maintained full activity for up to 6 hr of incubation. By contrast, in the presence of an actively working EthA, PyrG lost full activity within 4 hr when incubated with 7947882, and about 80% of its activity in 6 hr when incubated with 7904688 (Figures 1A and 1C).

At the end of incubation, to remove EthA as well as any unbound compounds, PyrG was re-purified by Ni-NTA (nitrilotriacetic acid) chromatography and dialyzed. Whereas PyrG from the blank reaction preserved its activity, the enzyme incubated in the full reaction remained completely inactive. Moreover, in the UV-Vis spectrum of PyrG incubated with EthA and 7947882, an additional peak appeared at 330 nm (Figure 1B). This peak, which was not present in the PyrG spectrum from blank reactions without NADPH, is characteristic of 7947882, thus demonstrating that, in contrast to its prodrug, the EthA-activated metabolite is able to bind PyrG. Similarly, the spectrum of PyrG incubated with 7904688 showed the broad peak between 310 and 400 nm, typical of the compound; this peak was absent in the blank control (Figure 1D). These results demonstrated that the conversion of 7947882 and 7904688 by EthA leads to active inhibitors of PyrG.

### Identification of Active Metabolites of 7947882

EthA is known to catalyze the oxygenation of the thioamide moiety of ETH, leading to the formation of S-oxide and S-dioxide products (Vannelli et al., 2002), as well as the oxygenation of the sulfide group of methyl(*p*-tolyl)sulfide (Fraaije et al., 2004). Thus, it is conceivable that EthA might catalyze a similar reaction on the thiophene moiety of 7947882. To confirm this hypothesis, we attempted to identify the active metabolite(s) of the 7947882 prodrug after purification from the EthA reaction mixture.

Two main products (M1 and M2) were isolated and subjected to mass spectrometry analysis. The two isolated compounds showed *m*/*z* values of 293 and 277, respectively, which are in agreement with the S-dioxide and the S-monoxide derivatives of the 7947882 compound. Moreover, the fragmentation spectra of the metabolites showed a pattern similar to that of 7947882, in accordance with mono- and di-oxygenation of the thiophene sulfur atom of the substrate (Figures 1E and 1F). The partially purified metabolites were tested against PyrG protein and found to inhibit its enzymatic activity. Notably, the M1 product showed a higher degree of inhibition.

To better characterize the 7947882 metabolites, its S-dioxide derivative was chemically synthesized, giving rise to compound 11426026. The mass spectrum of 11426026 showed the same pattern as the M1 compound, confirming that M1 corresponds to the 7947882 S-dioxide derivative (Figures 1E and 1F). Therefore, the effects of 11426026 toward *M. tuberculosis* growth and toward PyrG activity were assessed. The MIC of 11426026 for *M. tuberculosis* H37Rv, *ethA*, and *pyrG* mutant strains was determined (Table S2). Wild-type *M. tuberculosis* and the *ethA* mutant were similarly sensitive to 11426026 (with MICs close to that of the parent compound 7947882), showing that 11426026 does not require activation by EthA, whereas the *pyrG* mutant strain was resistant, thus demonstrating that PyrG could be the target of this active metabolite.

Indeed, this was confirmed when the inhibitory activity of 11426026 for PyrG was assessed, since the compound was effective against the wild-type enzyme. Interestingly, the inhibitory effects were only found at subsaturating concentrations of ATP (IC_50_ 0.035 ± 0.002 mM in the presence of 0.2 mM ATP). Moreover, the compound was not active against the PyrG V186G mutant when tested under the same conditions. In fact, the estimated IC_50_ value was 44-fold higher than against the wild-type enzyme (1.5 ± 0.15 mM), at an ATP concentration of 1.5 mM, which corresponds to the *K*_m_ of the mutant for this substrate (Figure S1A).

This evidence confirms the hypothesis that 11426026 affects or binds at the ATP-binding site of PyrG, behaving as a competitive inhibitor with respect to ATP (*K*_i_ 0.010 ± 0.002 mM; Figures S1B and S1C). The high *K*_m_ value of the PyrG V186G mutant for ATP probably reflects the structural changes resulting from the mutation, which distorts the ATP-binding site and leading to an even lower affinity for the 11426026 derivative, thus explaining the resistance to this compound.

The same procedure was used to identify the metabolite(s) derived from 7904688. In this case only one metabolite was found, corresponding to 3-phenyl-*N*-[(4-piperidin-1-ylphenyl)carbamoyl]propanamide (Figure S2). This derivative likely arises from sequential EthA reactions on the sulfur atom of the carbamothioyl moiety (Chigwada et al., 2014). However, this last metabolite showed no effect on PyrG activity. It is conceivable that the active metabolite(s) of 7904688 might be an unstable intermediate, thus precluding its isolation.

### 7947882 Inhibition of PyrG Alters Nucleotide Metabolism in *M. tuberculosis*

**Figure 2:**
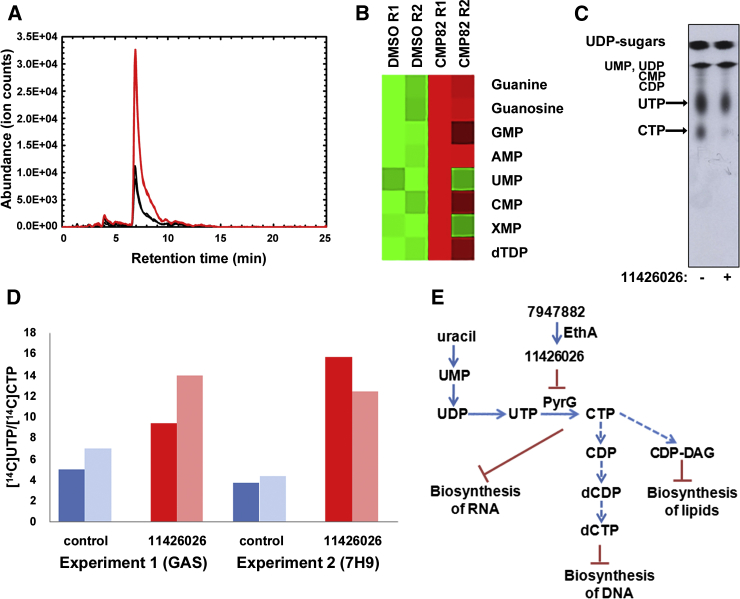
PyrG Inhibition Affects Nucleotide Metabolism in *M. tuberculosis* (A) Representative EIC for the AMP ion ((M + H)^+^, *m*/*z* = 348.07036) illustrating its increased pool size in 7947882-treated *M. tuberculosis* (red lines) compared with control (black lines). (B) Heatmap illustrating overall changes in nucleotide pool sizes in 7947882-treated *M. tuberculosis* compared with control. Data are derived from two biological replicates. (C) Thin-layer chromatography of nucleotide extract from [^14^C]uracil-labeled *M. tuberculosis* H37Ra grown on glycerol-alanine-salts (GAS) medium. The figure is a representative image from three separate experiments. (D) Ratio of radioactivity incorporated to [^14^C]UTP and [^14^C]CTP. Nucleotides were extracted from [^14^C]uracil-labeled *M. tuberculosis* H37Ra grown on GAS, or 7H9 media and separated by HPLC. The two columns represent duplicate 2-ml samples removed from the radiolabeled culture, processed separately. (E) Schematic representation of the major effects of PyrG inhibition in mycobacteria. See also Figure S3.

Since PyrG is a key enzyme involved in de novo pyrimidine biosynthesis (Meng et al., 2004), the effect of 7947882 on *M. tuberculosis* nucleotide metabolism was investigated. For this purpose, metabolomic experiments were performed with *M. tuberculosis* exposed for 24 hr to 7947882 (5× MIC) or its solvent, DMSO. Polar metabolites were extracted and analyzed by standard methods (de Carvalho et al., 2010; Larrouy-Maumus et al., 2013) that focused on bases, nucleosides, and nucleotides. *M. tuberculosis* H37Rv cells treated with 7947882 showed a substantial increase in the abundance of all nucleotide intermediates that were detected. Figure 2A illustrates extracted ion chromatograms (EIC) obtained for AMP in *M. tuberculosis* extracts treated with either compound or DMSO alone. Compound-induced changes in abundances of the ions detected are shown in Figure 2B. Taken together, these data demonstrate that direct inhibition of PyrG decreased CTP levels, leading to disruption of the nucleotide metabolic network, characterized by increased levels of several intermediates in the biosynthesis of pyrimidines and purines.

The molecular target of thiophenecarboxamides in mycobacteria was further corroborated through metabolic studies with [^14^C]uracil and the active metabolite 11426026, using *M. tuberculosis* H37Ra (MIC 4 μg/ml) grown in glycerol-alanine-salts (GAS) medium with or without 11426026 (16 μg/ml) for 1 hr, then [^14^C]uracil was added and radiolabeling continued for 3 hr. In the cells [^14^C]uracil is initially incorporated into [^14^C]uridine monophosphate (UMP) through the action of uracil phosphoribosyltransferase (Upp) from the pyrimidine salvage pathway (Villela et al., 2011). This is then further metabolized to the whole range of nucleotides and sugar nucleotides originating from uracil. After labeling, the cells were harvested and the nucleotide pool was extracted with diluted formic acid (Bochner and Ames, 1982). In the pilot experiment the PyrG substrate [^14^C]UTP was separated from the PyrG product [^14^C]CTP by thin-layer chromatography (TLC). An autoradiograph produced from the TLC plate clearly showed a decrease of [^14^C]CTP relative to [^14^C]UTP in treated *M. tuberculosis* compared with the control (Figure 2C). To quantify the changes, the labeling experiment was repeated under the same conditions and the nucleotides were analyzed by high-performance liquid chromatography (HPLC). Individual fractions co-eluting with the set of standards comprising UMP, uridine diphosphate (UDP), UTP, cytidine monophosphate, cytidine diphosphate, CTP, UDP-Gal, UDP-GlcNAc, and UDP-MurNAc pentapeptide were collected and quantified by measuring their radiolabel levels. Although incorporation of radioactivity into [^14^C]UTP and [^14^C]CTP was rather low in this experiment, the ratio of [^14^C]UTP/[^14^C]CTP did increase in the treated culture, as expected for PyrG inhibition (Figure 2D). Higher incorporation of [^14^C]uracil was achieved by using 7H9/ADC/Tween medium, thereby confirming the trend of increased [^14^C]UTP/[^14^C]CTP following 11426026 treatment (Figure 2D; Figure S3).

In conclusion, these experiments highlighted that inhibition of PyrG affects nucleotide metabolism and, thus, very likely several aspects of mycobacterial physiology. In particular, the metabolic changes should interfere not only with DNA and RNA biosynthesis, but also with other metabolic processes that require nucleotides, such as fatty acid, carbohydrate and amino acid biosynthesis, cell wall biosynthesis, and cAMP- and c-di-AMP-dependent signaling (Figure 2E).

### Validation of PyrG Essentiality In Vivo and Ex Vivo

**Figure 3:**
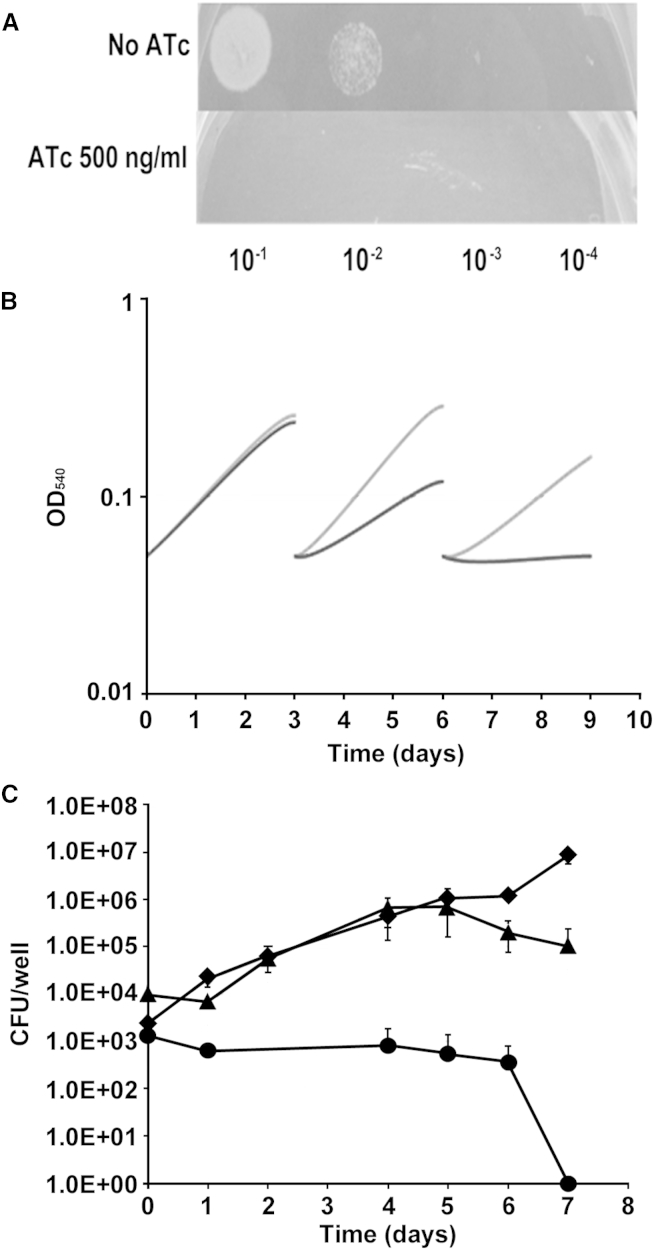
Essentiality of *pyrG* In Vitro and Ex Vivo (A) Ten microliters of a *pyrG* cKD mutant suspension containing about 10^5^ cfu were spotted at the indicated dilutions on Middlebrook 7H10 plates (±500 ng/ml ATc). (B) Bacteria were grown in 7H9 medium (±500 ng/ml ATc) and diluted 1:10 in fresh media (±500 ng/ml ATc) every 3 days. OD_540 nm_ was recorded and used to compile the growth curves. Each experiment was repeated at least twice. Gray line, *pyrG* conditional mutant grown without ATc; black line, *pyrG* conditional mutant grown with ATc. (C) Growth of *pyrG* conditional mutant and its parental strain (control) in THP-1-derived macrophages at an MOI of 1:20 (bacteria/macrophage). The results are expressed as cfu per well. The reported values represent the average and the SE obtained from two parallel independent infections. The experiment was repeated twice using independent bacterial inocula and THP-1 cultures. ATc (200 ng/ml) was added or not to the cell culture medium. Circles, *pyrG* conditional mutant plus ATc; diamonds, control plus ATc; triangles, *pyrG* conditional mutant, no ATc.

Since PyrG inhibition by the active metabolite of 7947882 has been unambiguously demonstrated, its validation as a drug target was further investigated. To show the essentiality of *pyrG* in *M. tuberculosis*, a conditional mutant was constructed where the *pyrG* promoter was replaced by the repressible promoter P_*ptr*_ in a strain carrying the TetR-PipOFF repressible system (Boldrin et al., 2010). In this conditional mutant, the expression of *pyrG* was expected to be downregulated by the addition of anhydrotetracycline (ATc) to the culture medium, thus leading to depletion of its protein product. The growth of the *pyrG* conditional mutant was evaluated on solid 7H10 and in liquid 7H9 media (± ATc, 500 ng/ml). In each case, this conditional mutant exhibited inhibition of growth upon ATc exposure, while its parental strain was not affected, thus clearly demonstrating that PyrG is essential for *M. tuberculosis* growth in vitro (Figures 3A and 3B).

PyrG essentiality was also verified during intracellular growth. For this purpose, THP-1-derived macrophages were infected with the *pyrG* conditional mutant or with its parental strain, and the cells were incubated in the presence or absence of ATc (200 ng/ml). While the control was able to divide intracellularly under both conditions, the *pyrG* conditional mutant grew similarly to the control only in the absence of ATc. When *pyrG* expression was downregulated by ATc, the number of viable bacteria dropped rapidly, demonstrating *pyrG* essentiality also during intracellular growth (Figure 3C). Proof that PyrG is essential both in vitro and ex vivo further corroborates the value of this enzyme as a drug target.

### PyrG Crystal Structure

**Figure 4:**
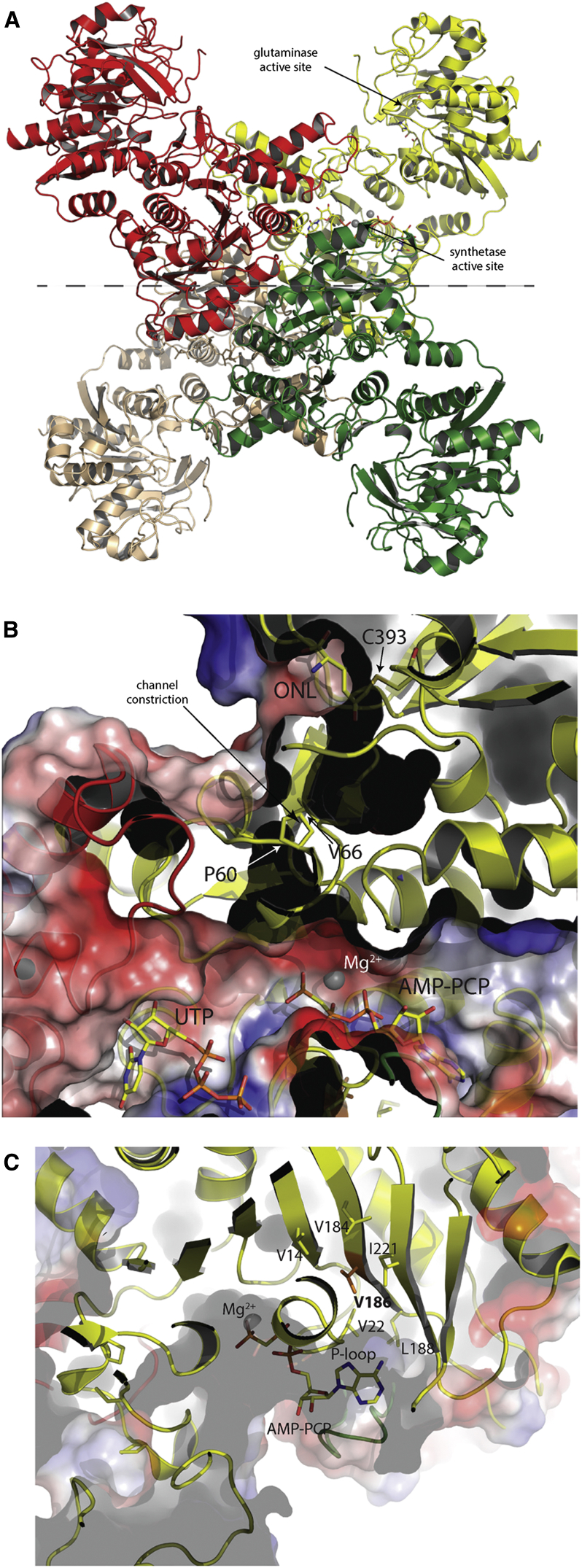
Crystal Structure of *M. tuberculosis* PyrG (A) Tetrameric structure of *M. tuberculosis* PyrG in complex with nucleotides and analogs, i.e. either UTP or UTP/AMP-PCP/L-DON. As observed in the other available crystal structures of CTP synthetases, the N-terminal synthetase domain is positioned at the center of the tetramer while the C-terminal glutaminase domain is pointing outwards. The gray dashed line indicates the PyrG dimers (yellow/red versus green/brown) as they can be found in the apo structure. (B) Surface “cut-through” of the synthetase active site, with UTP- and ATP-binding pockets, and “cut section” that shows a possible NH_4_^+^ channel connecting the glutaminase to the synthetase site. The electrostatic surface potential has been calculated and rendered by PyMol (Schroendinger, http://www.pymol.org). (C) Surface view of ATP-binding pocket, occupied by AMP-PCP, to show the location of Val186 (orange) mutated in Gly in the *M. tuberculosis*-resistant strains. Side chains of hydrophobic residues surrounding Val186 are depicted as sticks. See also Table S3; Figures S4 and S5.

The crystal structure of PyrG was solved by molecular replacement on a 2.0-Å resolution data set (Table S3). This structure showed a bidomain enzyme with an N-terminal amidoligase (ALase) domain, also commonly known as the synthetase domain (residues 1–278), connected through an interdomain linker (residues 279–Pro298) to a C-terminal glutamine amidotransferase (GATase) domain (residues 299–552), both domains displaying a Rossmann-like fold (Figure 4A). This bidomain architecture is typical of amidotransferases, already observed in the other available structures of full-length bacterial CTP synthetases (Goto et al., 2004; Endrizzi et al., 2004, 2005; Lauritsen et al., 2011). On the other hand, the 34-residue C-terminal extension of *M. tuberculosis* PyrG, which has no predicted secondary structure or known function, could not be traced due to the lack of supporting electron density, suggesting a high degree of flexibility.

The enzyme, in complex with either UTP, at 2.0-Å resolution, or UTP plus the non-hydrolyzable ATP analog AMP-PCP and the glutamine analog 5-oxo-L-norleucine (3.5 Å; Table S3), is a homotetramer with crystallographic 222 symmetry (Figure 4A), consistent with previous studies reporting positive cooperativity for ATP and UTP due to nucleotide-driven tetramerization. Indeed, another structure of the enzyme in the apo form at lower resolution (3.5 Å; Table S3), revealed a homodimeric protein, each homodimer representing half of the functional tetramer and showing a dimerization surface of ∼1,350 Å^2^ per monomer (Figure 4A), all in good agreement with previous structural studies (Endrizzi et al., 2004, 2005; Goto et al., 2004; Lauritsen et al., 2011). Also, consistent with the oligomeric assembly as a dimer of dimers being triggered by ATP/UTP, the nucleotide-binding pockets were delimited by residues from two (ATP) or three (UTP) different subunits (Figures S4A and S4B). Surprisingly, in the highest-resolution structure available (Table S3), UTP was found lying in both pockets, a likely artifact due to the high concentration of the nucleotide (5 mM) in the co-crystallization conditions (Figure S4C). It should be noted that the UTP orientation in the substrate-binding pocket is unproductive for the course of the reaction, as the pyrimidine ring points away from ATP. Moreover, this UTP orientation coincides with the CTP orientation observed in *E. coli* PyrG in complex with CTP and ADP (PDB: 2ad5; Figure S4C), suggesting that this structure likely represents an inhibited enzyme (Endrizzi et al., 2005).

In contrast, in the independent crystal form, grown in the presence of AMP-PCP as well as UTP and the glutamine analog 6-diazo-5-oxo-L-norleucine (L-DON), AMP-PCP lies in the ATP-binding site, as expected, with UTP maintaining the same orientation as above (Figures 4B and S4B). In addition, a covalent adduct was observed between the Cys393 sulfur and oxonorleucine, as expected from the reaction with L-DON (Hart and Powers-Lee, 2008), therefore confirming the role of Cys393 as the catalytic nucleophile within a Gln-hydrolyzing triad that includes His524 and Glu526 (Figures S4D and S4E). In agreement with similar observations made on *E. coli* PyrG (Endrizzi et al., 2004), a putative ammonia diffusion channel was visualized connecting the glutaminase active site in the C-terminal domain to the synthetase site at the N-terminal domain (Figure 4B). However, the tunnel is not continuous, but appears to be blocked by the side chains of residues Pro55, Pro60, and Val66, all located on the long β2 to β3 linker that includes the short α2 (Figures S4D and S4E), forming a constriction in the channel (Figure 4B). The residue Val186 mutated to Gly in the *M. tuberculosis*-resistant mutants lies on the β7 strand behind the conserved P loop (Gly16 to Gly25) that contributes to bind the ATP phosphates, its side chain being at closest ∼7 Å from the AMP-PCP β-phosphate (Figure 4C). Despite being located in the proximity of the ATP-binding pocket, the Val186Gly substitution does not provide any obvious explanation for the resistance profile to an ATP competitive inhibitor. Moreover, this mutation should have a destabilizing effect on the P loop and on its proper positioning within the ATP-binding cleft, leading to a decreased affinity for ATP. This hypothesis, suggested by the observation that the Val186 side chain is situated in a hydrophobic pocket delimited by Val14, Leu22 (belonging to the P loop), Leu184, Leu188, and Ile221 (Figure 4C), is further supported by the steady-state kinetics analysis of the V186G mutant enzyme reported above.

The availability of the *M. tuberculosis* PyrG structure is useful for both structure-activity relationship (SAR) studies and in silico docking approaches to find new PyrG inhibitors that do not require EthA activation.

### SAR Study of 7947882

**Figure 5:**
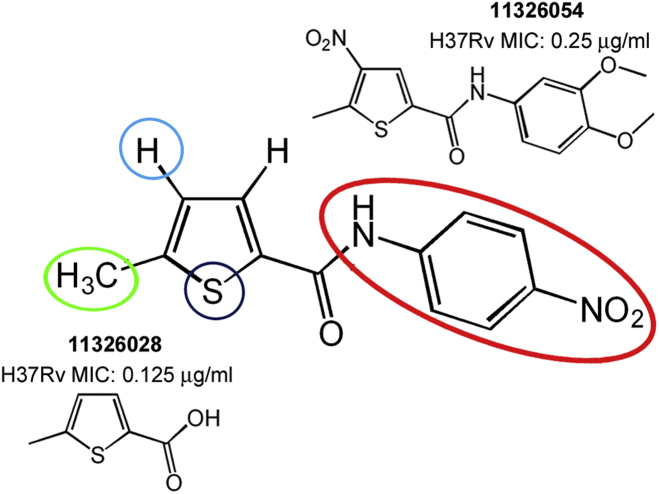
SAR Optimization Strategy of 7947882 Compound Modifications made on the thiophene ring (positions 4, 5 and the sulfur atom) and the 4-nitroaniline moiety led to two more active compounds (11326028 and 11326054). See also Tables S4 and S5.

To improve the antitubercular activity of 7947882, and to understand the substituent requirements needed to achieve activity against *M. tuberculosis*, SAR studies were performed (Figure 5). 105 derivatives of compound 7947882 were synthesized and tested for their activity against *M. tuberculosis* H37Rv. The substitutions concerned mainly the thiophene ring and the 4-nitroaniline moiety.

Substitution of the thiophene ring (with furan, pyrazole, or methylthiazole) led to inactive compounds (Table S4A). The substitution of the 4-nitroaniline led, in general, to decreased potency of the compounds (Table S4B). The presence of the *p*-nitro group was associated with the best MIC, but was not strictly required, since its substitution with halogen atoms or a methyl group caused only a small increase in MIC. Moreover, the addition of further substituents to the other positions of the phenyl ring did not improve efficacy. For instance, introduction of bulkier substituents, such as S-methyl or sulfonamide, or the substitution of the aniline with formimidamide derivatives, was even detrimental for activity, as these compounds all showed a higher MIC (Table S4B).

Furthermore, no improvement arose from modification of the substituents in the thiophene moiety. Lack of the methyl group in position 5 of the thiophene led to an increase in the MIC (Table S4C), as did introduction of a methyl or nitro group in position 4, with the exception of compound 11326054 which showed a lower MIC value (0.25 μg/ml) (Figure 5; Tables S4D and S4E). Curiously, analogs of this compound lacking the nitro group (11326008) did not show lower MIC (2 μg/ml). Moreover, all compounds with the thiophene moiety substituted with a nitro group showed lower potency than those lacking this group. Such high activity of compound 11326054 is conceivably connected with the antimycobacterial properties of nitrothiophenes (Hartkoorn et al., 2014), and in parallel with the amide moiety discussed in this paper.

Finally, substitution of the aniline with a hydroxy group, to give the 5-methylthiophene-2-carboxylic acid (11326028), led to the most potent compound (MIC of 0.128 μg/ml, Figure 5). The carboxylic group of compound 11326028 was fundamental for its antimycobacterial activity, since the carboxamide derivative was less active and, likewise for compound 7947882, modification of the thiophene substituents led to less active derivatives (Table S4F).

The *M. tuberculosis pyrG* mutant 88.7 showed levels of resistance to both 11326028 and 11326054 derivatives that were significantly higher (MIC values >8 μg/ml) compared with that of the wild-type strain, thus confirming that they still target PyrG. Similarly, the 81.10 mutant (mutated in *ethA* gene) was resistant to both compounds, indicating that they still need to be activated by EthA. This result was confirmed by the fact that the compounds are substrates of the enzyme (*k*_cat_ values of 1.33 ± 0.02 min^−1^ and 0.98 ± 0.03 min^−1^ for 11326028 and 11326054, respectively).

Finally, five derivatives of the active EthA metabolite 11426026 were synthesized. These compounds were all active toward wild-type PyrG, but not against the V186G mutant. However, none of these compounds showed an improved MIC compared with the active metabolite of 7947882 (Table S5).

### Docking of the 11426026 Active Metabolite and PyrG Inhibitors

To acquire insight into the binding between the active metabolite of 11426026 and PyrG, a careful computational analysis of the possible poses of the compound was performed. Docking the 11426026 compound demonstrated that it would only successfully dock in the PyrG ATP site (Figures S5A, S5B and S5C). The superimposition with the UTP molecule shows a partial overlap. The phenyl ring is suggested to pi-stack with Arg223 while the nitro group is proposed to interact with Ala253 and Asp252.

Similarity searching based on the 4-nitroacetanilide portion of the molecule resulted in 12 similar compounds present in the Collaborative Drug Discovery (CDD) database (Ekins and Bunin, 2013; Ananthan et al., 2009; Ekins et al., 2014; Maddry et al., 2009; Reynolds et al., 2012). Four of these compounds were tested in vitro against PyrG enzymatic activity. One compound, CDD-823953 (LibDock score 106.7), was a weak PyrG inhibitor (*K*_i_ = 88.9 μM). Figures S5D and S5E show how this compound may bind less optimally in the ATP-binding site with the 4-nitroacetanilide portion in a different position to that seen with 11426026. Docking of compounds may be instructive for SAR until the co-crystal structure with a ligand is obtained. For example, the 11326054 sulfone was also docked in the PyrG structure and was shown to be in an orientation similar to that of the 11426026 active metabolite (Figures S5F and S5G).

## Significance

**New leads and new targets are required for tuberculosis drug development. Using phenotypic screening of a chemical library, two thiophenecarboxamide derivatives were identified that inhibited *M. tuberculosis* under replicating, non-replicating, and intracellular growth conditions. Both compounds were activated by the EthA monooxygenase, and the main metabolite of one of them (7947882), identified by mass spectrometry, was shown to target the CTP synthetase PyrG. The active metabolite was synthesized and shown to behave as a competitive inhibitor toward the ATP-binding site of PyrG, thus validating this enzyme as a new antitubercular drug target. Further validation was obtained genetically using conditional knockdown of *pyrG* to prove its essentiality in all the physiological states. A metabolomic approach demonstrated that the thiophenecarboxamide derivatives caused general deregulation of nucleotide metabolism, consistent with the inhibition of CTP synthetase. The combined evidence strongly indicates that PyrG is potentially a clinically relevant drug target. To overcome the requirement for EthA activation, we obtained high-resolution crystal structures of PyrG to underpin structure-based drug design. This approach has already generated additional lead compounds that inhibit this new drug target under all conditions tested.**

## Experimental Procedures

### NIAID Library Screening

CB2, a library of 594 compounds selected from an HTS screen on *M. tuberculosis* H37Rv (Ananthan et al., 2009; Goldman and Laughon, 2009; Maddry et al., 2009), was kindly provided by NIAID (Prof. R.C. Goldman). These compounds were initially screened at 10 μg/ml in duplicate for activity against H37Rv and ss18b in 96-well format, using the resazurin reduction microtiter assay. Compounds with a percentage of inhibition of H37Rv growth of more than 80% were subsequently analyzed for their MIC, intracellular activity against H37Rv, and cytotoxicity against the human hepatocellular carcinoma cell line HepG2 and Huh7, the human lung epithelial cell line A549, and the murine macrophage cell line RAW 264.7 (see Supplemental Experimental Procedures).

### Compounds Used and Synthesis of Their Derivatives

7904688 and 7947882 were purchased from ChemBridge Corp (http://www.chembridge.com/index.php). Synthetic routes of thiophene derivatives, experimental details, and compound characterization data are provided in the Supplemental Experimental Procedures.

### Isolation and Characterization of *M. tuberculosis* Mutants Resistant to 7947882 and 7904688

The isolation of *M. tuberculosis* mutants was performed by plating ∼10^10^ cells from an exponential growth phase wild-type culture onto 7H11 medium containing different concentrations of 7947882 and 7904688, ranging from 5- to 20-fold the MIC of the wild-type strain.

Genomic DNA of *M. tuberculosis*-resistant mutants and wild-type strain was isolated and sequenced by using Illumina HiSeq2000 technology at IGA Technology Services (Udine, Italy). For the bioinformatic analysis of Illumina data, repetitive PE and PPE gene families were discarded as well as SNPs and indels with less than 50% probability. The mutations found in *ethA* (*Rv3854c*) and *pyrG* (*Rv1699*) (http://tuberculist.epfl.ch/) were confirmed by Sanger sequencing (Eurofins MWG Operon), after PCR amplification using the oligonucleotides presented in the Supplemental Information. PCR products were purified using the Wizard SV Gel and PCR Clean-Up system (Promega).

### Overexpression of *ethA* in *M. tuberculosis* H37Rv

*M. tuberculosis ethA* was cloned into pSODIT-2, a shuttle expression vector containing the hygromycin resistance determinant, after PCR amplification using primers indicated in the Supplemental Information, *Pfu* DNA Polymerase (Promega), and genomic DNA as template. PCR fragments were digested with *Bam*HI and *Hin*dIII and ligated to the pSODIT-2 generating pSODIT/*ethA*. *M. tuberculosis* H37Rv competent cells were transformed with pSODIT-2 or pSODIT/*ethA*, and plated onto complete Middlebrook 7H11 agar plates supplemented with 20 μg/ml hygromycin and different concentrations of 7947882 or 7904688, ranging from 0.125 to 20 μg/ml.

### Enzyme Production and Characterization

*M. tuberculosis* PyrG and EthA were obtained in recombinant forms in *E. coli* and purified by standard methods. Enzymatic assays were performed according to the published methods (Fraaije et al., 2004; Lunn et al., 2008). See also Supplemental Experimental Procedures.

### Construction of a *M. tuberculosis pyrG* Knockdown Mutant

The first 714 bp of *pyrG* coding sequence was amplified using RP1609 and RP1610 primers and cloned in the suicide plasmid pFRA170 downstream of a P_*ptr*_-derived promoter. To replace *pyrG* promoter with P_*ptr*_, 10 μg of this plasmid was used to transform TB38, an H37Rv derivative harboring the TetR-PipOFF system in its genome at the L5 *attB* site (Boldrin et al., 2010). Selection of recombinants was achieved using 7H10 agar plates containing hygromycin (50 μg/ml). Integration of the suicide plasmid was confirmed by PCR. Since *pyrG* might be co-transcribed with its downstream genes (*rv1700-rv1701*), the latter genes were provided in *trans* on a pMV261-derived plasmid. In this way the final *pyrG* conditional knockdown (cKD) strain was obtained. This cKD strain was used for *pyrG* essentiality evaluation in both in vitro and ex vivo experiments (see Supplemental Experimental Procedures).

### PyrG Crystallization, Data Collection, and Structure Determination

Crystallization screenings of PyrG in the presence of various ligands were carried out at 18°C by sitting drop in 96-well format (200 + 200 nl drops) with a Mosquito dispensing robot (TTP Labtech). Crystals were identified in several conditions. PyrG in apo form: 10% PEG8000, 200 mM Ca acetate, 100 mM HEPES (pH 7.5); complex with UTP: 17% PEG20000, 100 mM MgCl_2_, 100 mM Tris-HCl (pH 8.5); complex with AMP-PCP, UTP, and L-DON: 30% PEG2000 MME, 100 mM NaCl, 100 mM bicine (pH 9.0). All data sets were collected on the Proxima-1 beamline at the Soleil synchrotron (Gif-sur-Yvette, France) from single crystals at 100 K, processed with XDS (Kabsch, 2010) and merged with Aimless from the CCP4 suite (Winn et al., 2011). The structure was first solved by molecular replacement with the program MOLREP (Murshudov et al., 1997) on a data set collected from a PyrG-UTP crystal, using the structure of *Thermus thermophilus* CTP synthetase in complex with sulfate (PDB: 1vcn; Goto et al., 2004) as the search model. Refinement was carried out with Refmac5 (Murshudov et al., 2011) or autoBUSTER (Bricogne et al., 2011). The other data sets were solved by molecular replacement with MOLREP and the coordinates of a partially refined *M. tuberculosis* PyrG structure as the search model. Final models were validated through the Molprobity server (Chen et al., 2010). Docking of PyrG inhibitors was performed as described in the Supplemental Experimental Procedures.

### In Vitro EthA Metabolite Production and Identification

For in vitro EthA metabolite production, 30 mg of 7947882 was incubated with 10 mg of EthA in 50 mM potassium phosphate (pH 8.0), 500 μM NADPH, 10 μM BSA, at 37°C for 5 hr under agitation. To produce the PyrG-EthA metabolite complex, the EthA reaction was performed in the presence of 45 μM PyrG. Reaction products were purified and analyzed as described in Supplemental Experimental Procedures.

### Metabolomic Experiments

Experimental Procedures have been described elsewhere (Larrouy-Maumus et al., 2013; Brauer et al., 2006). In brief, *M. tuberculosis* H37Rv was grown initially in 7H9 (with 0.5 g/l BSA, 0.05% tyloxapol, 0.2% glycerol, 0.2% glucose, and 0.085% NaCl) until late logarithmic phase (OD_600_ = 1.0) and 1 ml was layered onto 22-mm nitrocellulose filters (0.22 μm) under vacuum filtration. *M. tuberculosis*-laden filters were placed atop 7H10 (supplemented as 7H9) and incubated at 37°C for 5 days, after which the filters were transferred to 7H10 containing 7947882 (5× MIC = 2.5 μg/ml) or the control. After 24 hr, the bacteria were metabolically quenched by plunging *M. tuberculosis*-laden filters into acetonitrile/methanol/H_2_O (2:2:1) pre-cooled to −40°C. The metabolites were extracted by mechanical lysing of the *M. tuberculosis*-containing solution with 0.1-mm zirconia beads. Lysates were clarified by centrifugation, filtered, and metabolites analyzed by liquid chromatography-mass spectrometry as described in Supplemental Experimental Procedures.

For metabolic labeling of *M. tuberculosis* H37Ra with [^14^C]uracil, 10 ml of the GAS medium (Takayama et al., 1975) was inoculated in the ratio 1:100 with an *M. tuberculosis* H37Ra pre-culture grown in Sauton medium. After 7 days of static growth at 37°C, the culture was split into two aliquots, and 11426026 (final concentration of 16 μg/ml) was added to one and DMSO to the other as a control. After 1 hr, [^14^C]uracil (American Radiolabeled Chemicals, specific activity 53 mCi/mmol) was added to a final concentration 1 μCi/ml. Radiolabeling was carried out for 3 hr, then two 2-ml batches were removed from each culture. The bacteria were harvested by centrifugation, washed twice with a cold physiologic solution, and immediately extracted with 110 μl of ice-cold 9% (v/v) formic acid for 30 min. The formic acid extract was recovered by centrifugation and its radioactivity was quantified by scintillation spectrometry in 5 ml of EcoLite scintillation liquid (MP Biomedicals). The nucleotide extract was stored at −20°C and was typically analyzed by TLC or HPLC within 24 hr (see Supplemental Experimental Procedures). Alternatively, radiolabeling was performed as above with *M. tuberculosis* H37Ra culture grown to OD_600_ = 0.375 in 7H9 medium at 37°C.

## Author Contributions

R.C.H. performed the screening of NIAID library procured by S.T.C.; G.M., A.L.dJ.L.R., and M.R.P. isolated and characterized the resistant mutants; M.F. performed bioinformatics analysis of Illumina data; G.M., L.R.C., M.E., and N.B. performed cloning, and protein expression and purification; L.R.C. and M.E. performed enzymatic assays; L.R.C., M.E., and A.P. performed metabolite isolation and identification; V.M., E.K., and A.L. performed derivative synthesis; G.D. and F.B. performed studies on *pyrG* essentiality; L.B.M., I.C., Z.S., and J.B. performed metabolomic studies; M.B. performed crystallographic studies; S.E. and M.B. performed docking experiments; R.F., G.Z., V.M., R.M., L.P.S.C., A.R.B., K.M., P.M.A, G.R., S.T.C., and M.R.P. supervised and directed the work; L.R.C., M.B., S.E., R.F., G.Z., V.M., R.M., L.P.S.C., A.R.B., K.M., G.R., S.T.C., and M.R.P. wrote the paper. All authors discussed the results and commented on the manuscript.

## Figures and Tables

**Table 1:** Activity In Vitro in Latent and Replicating *M. tuberculosis* Growth and Activity Ex Vivo of the Two Selected Compounds

Compound ID	Structure	H37Rv MIC (μg/ml)	ss18b IC_50_/IC_90_ (μg/ml)	Intracellular IC_50_/IC_90_ (μg/ml)
7904688		0.5	2.5/20	0.175/0.625
7947882		0.5	2.5/10	0.625/1.25

**Table 2:** Main Features of *M. tuberculosis* Mutants Resistant to 7947882 and 7904688

*M. tuberculosis* Strains	MIC (μg/ml)			WGS Sequencing Results (Amino Acid Change)	
	7947882	7904688	ETH	*ethA*	*pyrG*
H37Rv	0.5	0.5	1	–	–
82.14	>40	>40	10	T133C (W45R)	T557G (V186G)
82.19	>40	>40	10	T386C (L129P)	T557G (V186G)
82.22	>40	>40	10	ΔT-94	T557G (V186G)
88.7	5–10	10	0.5	–	T557G (V186G)
88.10	5–10	10	0.5	–	T557G (V186G)
81.10^a^	>40	>40	10	Δ1109–1137	–

See Table S1.

a Laboratory collection.

## Accession Numbers

The PDB accession numbers for coordinates and structure factors of the crystal structures described here are PDB: 4ZDI (PyrG in apo form); PDB: 4ZDJ (PyrG in complex with two molecules of UTP/Mg^2+^); and PDB: 4ZDK (PyrG in complex with UTP, AMP-PCP, Mg^2+^, and 5-oxo-L-norleucine).

## Supplemental Information

Document S1. Figures S1–S5, List of Oligonucleotide Primers, Tables S1–S3, S5, and Supplemental Experimental Procedures

Table S4. MIC Values of 7947882 Derivatives

Document S2. Article plus Supplemental Information
