# Supplementary material for: Thiophenecarboxamide Derivatives Activated by EthA Kill Mycobacterium tuberculosis by Inhibiting the CTP Synthetase PyrG
Source: Chem Biol. 2015 Jul 23;22(7):917–27. doi: 10.1016/j.chembiol.2015.05.016 (PMC4521081; doi:10.1016/j.chembiol.2015.05.016)
Supplement: Document S1. Figures S1–S5, List of Oligonucleotide Primers, Tables S1–S3, S5, and Supplemental Experimental Procedures [file mmc1.pdf]

Chemistry & Biology, Volume *22*

Supplemental Information

Thiophenecarboxamide Derivatives Activated by EthA Kill *Mycobacterium tuberculosis* by Inhibiting the CTP Synthetase PyrG

Giorgia Mori, Laurent R. Chiarelli, Marta Esposito, Vadim Makarov, Marco Bellinzoni, Ruben C. Hartkoorn, Giulia Degiacomi, Francesca Boldrin, Sean Ekins, Ana Luisa de Jesus Lopes Ribeiro, Leonardo B. Marino, Ivana Centárová, Zuzana Svetlíková, Jaroslav Blaško, Elena Kazakova, Alexander Lepioshkin, Nathalie Barilone, Giuseppe Zanoni, Alessio Porta, Marco Fondi, Renato Fani, Alain R. Baulard, Katarína Mikušová, Pedro M. Alzari, Riccardo Manganelli, Luiz Pedro S. de Carvalho, Giovanna Riccardi, Stewart T. Cole, and Maria Rosalia Pasca

SUPPLEMENTAL INFORMATION

Thiophenecarboxamide derivatives activated by EthA kill Mycobacterium tuberculosis by inhibiting the CTP synthetase PyrG

Giorgia Mori, Laurent R. Chiarelli, Marta Esposito, Vadim Makarov, Marco Bellinzoni, Ruben C. Hartkoorn, Giulia Degiacomi, Francesca Boldrin, Sean Ekins, Ana Luisa de Jesus Lopes Ribeiro, Leonardo B. Marino, Ivana Centárová, Zuzana Svetlíková, Jaroslav Blaško, Elena Kazakova, Alexander Lepioshkin, Nathalie Barilone, Giuseppe Zanoni, Alessio Porta, Marco Fondi, Renato Fani, Alain R. Baulard, Katarína Mikušová, Pedro M. Alzari, Riccardo Manganelli, Luiz Pedro S. de Carvalho, Giovanna Riccardi, Stewart T. Cole, and Maria Rosalia Pasca.

INVENTORY OF SUPPLEMENTAL INFORMATION

Figure S1 Inhibition of PyrG activity by 11426026. Related to Figure 1

Figure S2 Identification of in vitro EthA metabolite(s) of 7904688. Related to Figure 1

Figure S3 HPLC analysis of [^{14}C]-uracil-labeled nucleotide extracts from *M. tuberculosis* H37Ra grown in 7H9/ADC/Tween media. Related to Figure 2.

Figure S4 Representation of PyrG in complex with different ligands. Related to Figure 4.

Figure S5 Docking of PyrG inhibitors. Related to Figure 4.

List of oligonucleotide primers used in this study, related to Experimental Procedures

Table S1 Profile of resistance of *M. tuberculosis* cells overexpressing *ethA*. Related to Table 2.

Table S2 MIC values to 11426026 of *ethA* and *pyrG M. tuberculosis* mutant strains . Related to Figure 1.

Table S3 Crystallographic data collection and refinement statistics. Related to Figure 3.

Table S4 MIC values of 7947882 derivatives. Related to Figure 5. (Excel File)

Table S5 Biological and biochemical characterization of 11426026 derivatives. Related to Figure 5.

Supplemental Experimental Procedures

SUPPLEMENTARY FIGURES

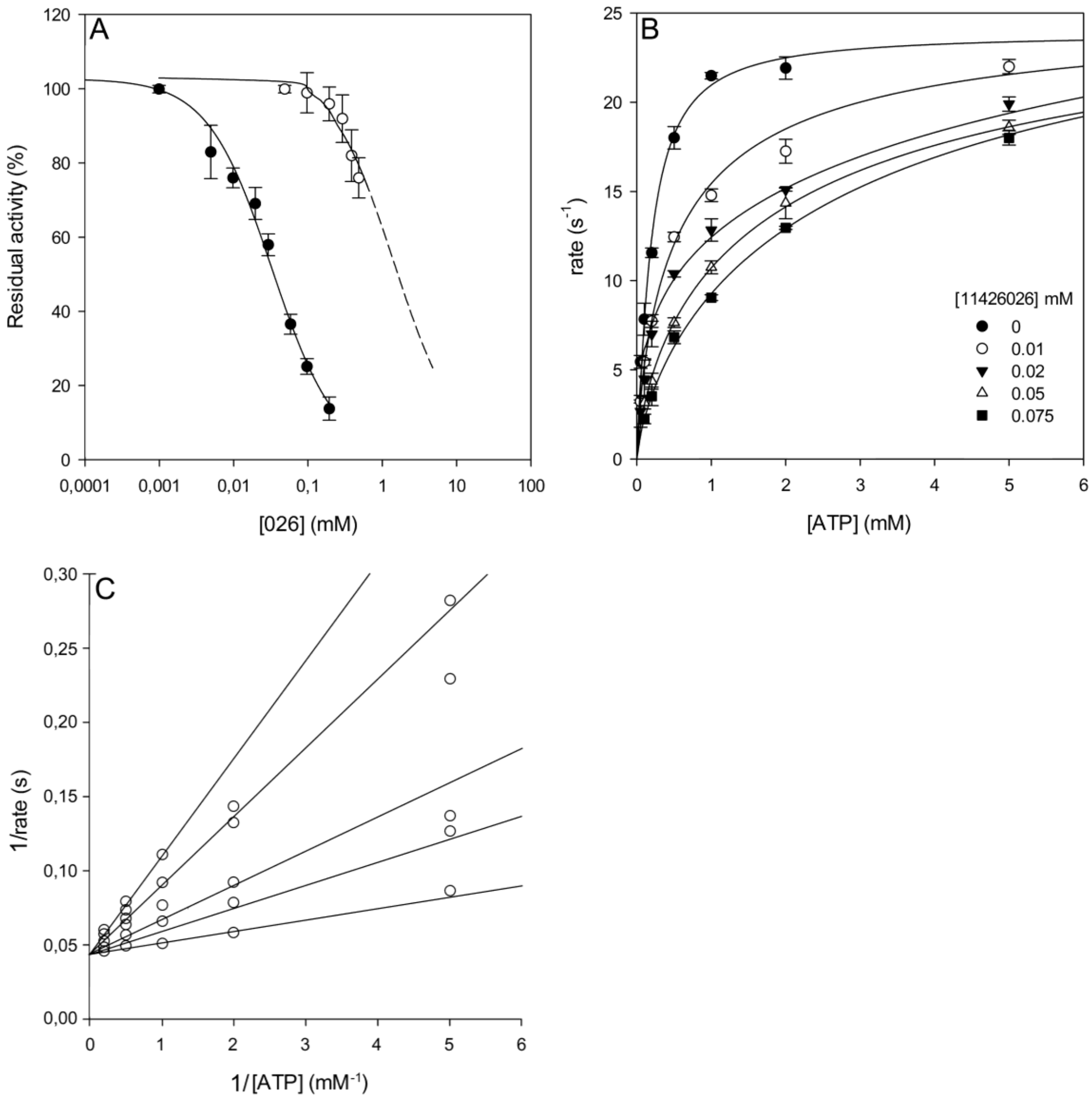

Figure S1 related to Figure 1. Inhibition of PyrG activity by 11426026 compound

(A) IC_{50} determination for 11426026 against wild type (closed symbols) and V186G mutant (open symbols) PyrG. IC_{50} values were determined at concentrations of ATP corresponding to the K_m values for each enzyme (0.2 mM for the wild-type and 1.5 mM for the mutant), and by fitting the experimental data as reported in Materials and Methods.

(B) Steady state kinetics analysis towards ATP of PyrG in the presence of different concentrations of 11426026 compound highlights the competitive behavior of the inhibitor.

(C) Global reciprocal plot of data in panel B.

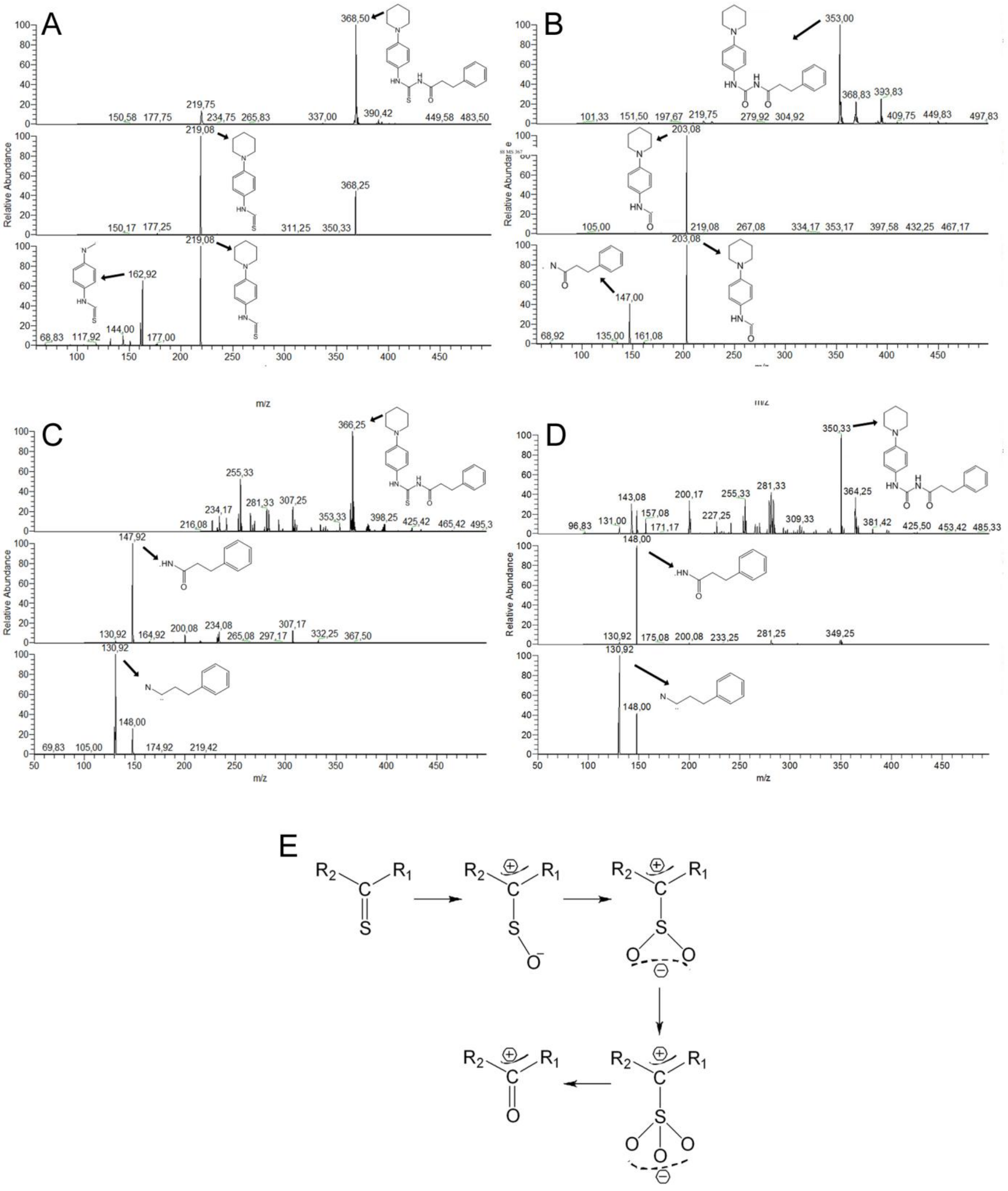

Figure S2, related to Figure 1. Identification of *in vitro* EthA metabolite(s) of 7904688

(A) Mass spectrometry analysis of the 7904688 in positive mode, MS2 and MS3 fragmentations.

(B) mass spectrometry analysis of the EthA metabolite of 7904688 in positive mode, MS2 and MS3 fragmentations.

(C) mass spectrometry analysis of 7904688 in negative mode, MS2 and MS3 fragmentations.

(D) mass spectrometry analysis of the EthA metabolite of 7904688 compound in negative mode.

(E) Hypothesized EthA mediated oxigenation reactions of 7904688, leading to the final metabolite.

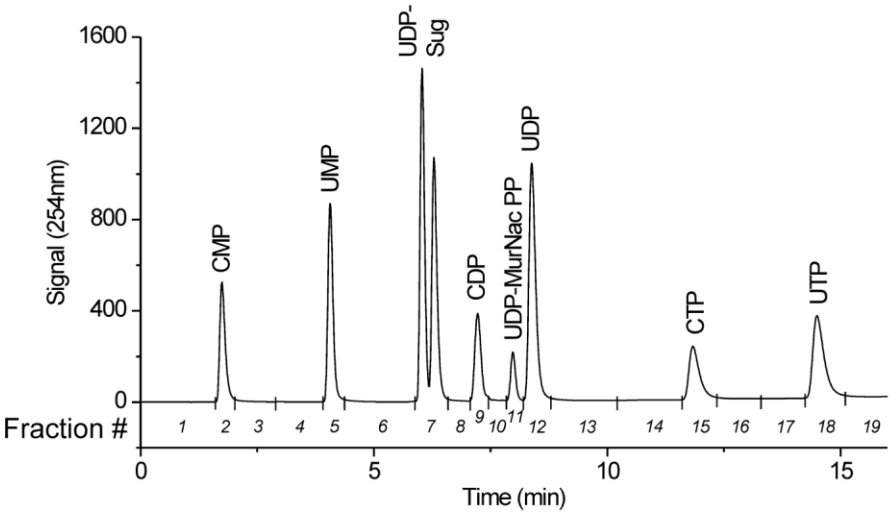

	Control		11426026		
Fraction #	A	B	A	B	
1	61	321	59	69	
2	2	4	2	8	CMP
3	0	0	0	0	
4	18	24	13	44	
5	991	888	1563	1358	UMP
6	128	44	29	43	
7	4323	3620	3418	3107	UDP-sugars
8	28	0	0	0	
9	422	344	22	7	CDP
10	12	72	37	41	
11	0	0	0	0	UDP-Mur-PP
12	302	288	220	257	UDP
13	1	0	0	0	
14	12	0	21	0	
15	362	319	155	168	CTP
16	2	0	0	0	
17	24	0	1	5	
18	1346	1384	2434	2089	UTP
19	64	45	49	42	

**UTP/CTP**	**3.7**	**4.3**	**15.7**	**12.4**

Figure S3 related to Figure 2. HPLC analysis of [^{14}C]-uracil-labeled nucleotide extracts from *M. tuberculosis* H37Ra grown in 7H9/ADC/Tween media

Approximately 7,000 dpm was loaded on HPLC and separated as described in Methods. The fractions were collected as shown in the chromatogram. Radioactivity in the individual fractions was quantified by scintillation spectrometry and is shown in the table. The experiment was performed with duplicate 2 ml samples (A, B) removed from each radiolabeled culture, which were processed and analysed separately.

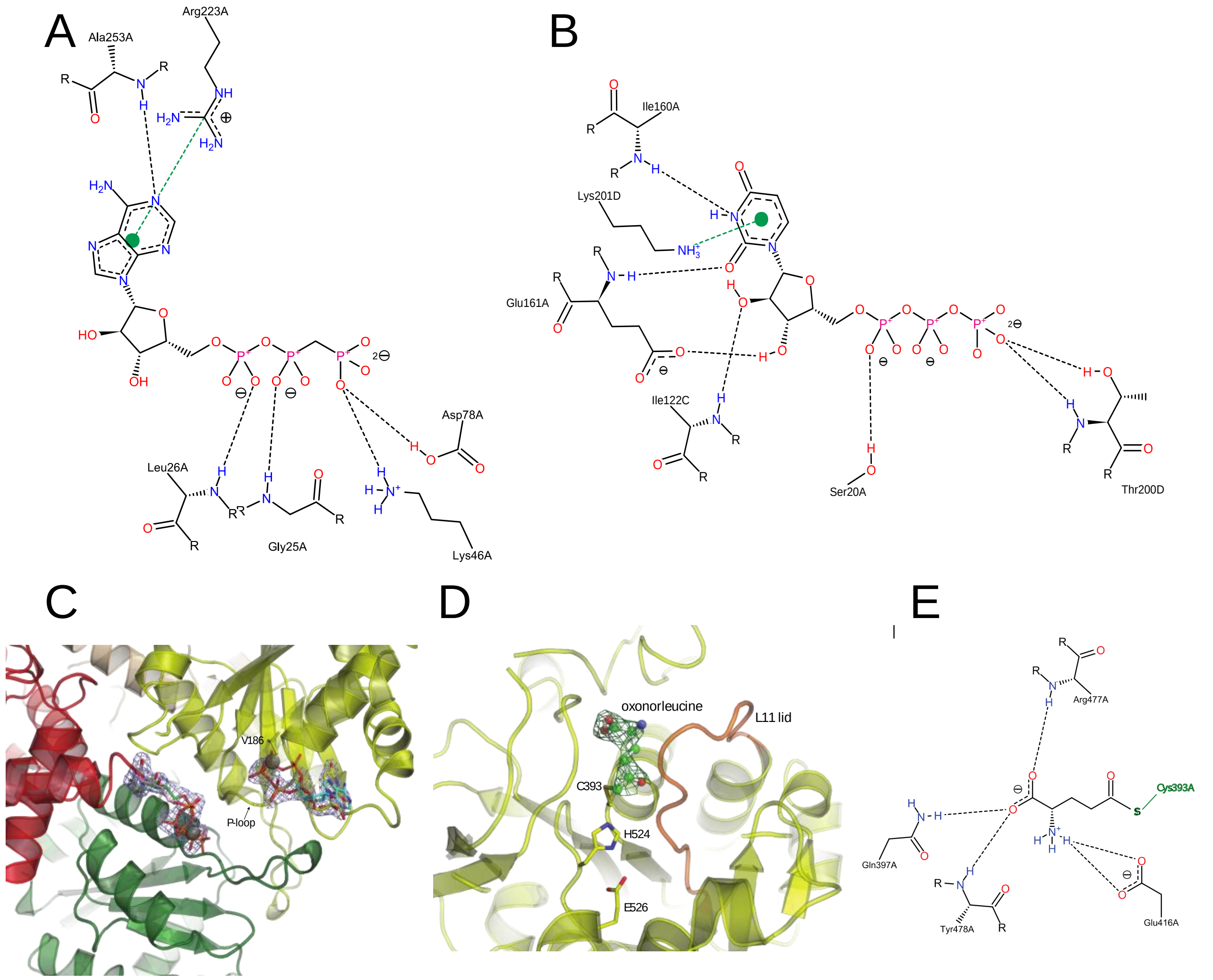

Figure S4, related to Figure 4. Representation of PyrG in complex with different ligands.
Bidimensional representation of ligand-enzyme interactions for AMP-PCP (A) and UTP (B). UTP occupies the CTP-binding pocket, similarly to the *E. coli* PyrG with CTP and ADP (PDB: 2ad5; Endrizzi et al., 2005). Figure is realized with Poseview server (http://poseview.zbh.uni-hamburg.de/; Stierand and Rarey, 2010).
(C) Cartoon view of the active site of *M. tuberculosis* PyrG in complex with UTP, showing two UTP molecules occupying the CTP and the ATP-binding pocket. The carbons of CTP binding site are in green (left), while the carbons of the ATP-binding pocket in yellow (right). Gray spheres represent Mg^{2+}. The corresponding $2F_o$-F_c electron density map, contoured at the 1σ level, is shown as a blue mesh. The lateral chain of Val186, mutated to Gly in the *M. tuberculosis* strain resistant to 7947882, is shown as sticks. Colors refer to the different protomers within the PyrG functional tetramer. Superimposed, and shown as sticks, are the CTP and ADP ligands in complex with *E. coli* PyrG (PDB: 2ad5), representing a CTP-inhibited enzyme (Endrizzi et al., 2005). Coordinates of the two orthologous enzymes superimpose with an rmsd=1.18 Å but, for clarity, only the ligands from 2ad5 following the superimposition are shown.
(D) View of the glutaminase active site in the C-terminal domain of PyrG with UTP (not shown) and 5-oxo-L-norleucine (after 30' soak with 25 mM). The figure represents the catalytic triad (Cys393-His524-Glu526) involved in glutamine hydrolysis in class I amidotransferases (Hart and Powers-Lee, 2008; Zalkin and Smith, 1998), and the key nucleophile Cys393 in alkylated form with 5-oxo-L-norleucine after the reaction with L-DON, which mimics the tetrahedral enzyme-Gln reaction intermediate. The green mesh represents Fourier difference F_o-F_c electron density before the covently-bound ligand was modeled. In orange, the 'L11 lid' including β14 and the loop that connects it to α14 (residues Val361 to Glu373 in PyrG; Fig. S5), proposed to fold over the Gln substrate in the glutaminase active site (Endrizzi et al., 2004).
(E) 2D depiction of the oxonorleucine interactions in the GATase active site, realized with the Poseview server.

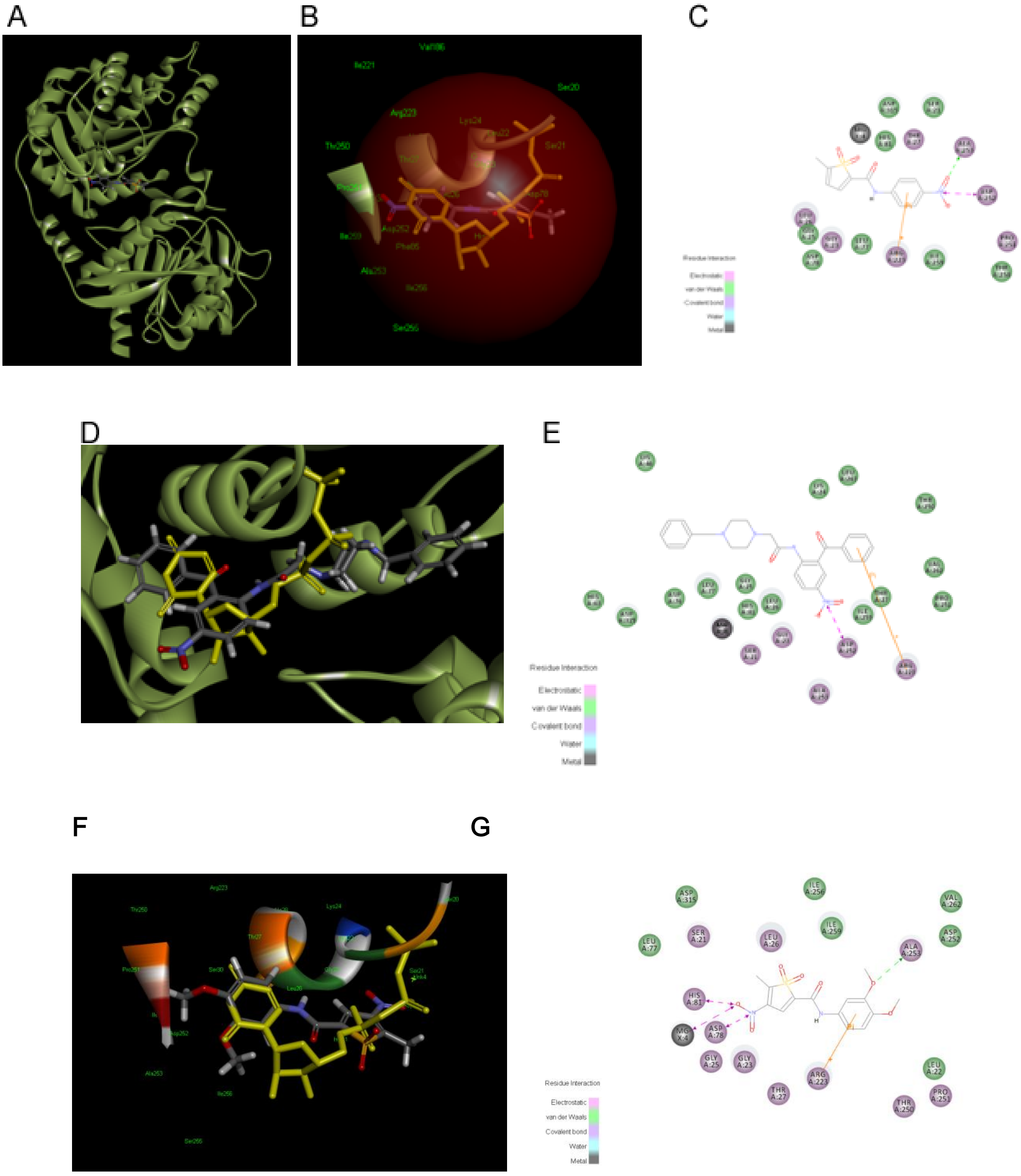

Figure S5, related to Figure 4. Docking of PyrG inhibitors.
(A) 11426026 docked in the PyrG ATP binding site.
(B) Overlap of 11426026 (colored by atom) and the UTP structure (yellow).
(C) 2D interaction map for 11426026.
(D) CDD-823953 docked in the ATP binding site (colored by atom) and the UTP structure (yellow).
(E) 2D interaction map for CDD-823953.
(F) Overlap of the 11326054 sulfone and the UTP structure.
(G) 2D interaction map for 11326054 sulfone.

SUPPLEMENTARY TABLES

List of oligonucleotide primers used in this study, related to Experimental Procedures.

Primers	Sequence (5'-3')	PCR product (bp)	Purpose
**PyrGseqF or**	AAATCGGGGGCACTGTCG	330	Sequencing of *pyrG*
**PyrGseqRev**	CCTTGGGTATGTCGTAGAT		
**EthAseqF or**	TGGCAGCTTACTACGTGTC	1647	Sequencing of *ethA*
**EthAseqRev**	CTGGGCGGGGTGACATTC		
**EthAsodF or**	TTggatccATGACCGAGCACCTC (*Bam*HI)	1470	Cloning of *ethA* in pSODIT-2
**EthAsodRev**	TTaagcttCTAAACCCCCACCGG (*Hind*III)		
**EthAsumF or**	ATGACCGAGCACCTCG	1470	Cloning of *ethA* from *M.tuberculosis* into pET-SUMO vector
**EthAsum Rev**	CTAAACCCCCACCGG		
**RP1609**	TTTTatgcatCGAAAGCACCCGCAAACC (*Nsi*I)	714	Construction of *pyrG* knock-down mutant
**RP1610**	TTTTactagtCATCAACGCAATCTTGTTTTTCA (*Spe*I)		
**MB319**	ATCAAGGATCCGAGAATCTTTATTTTCAAGGA**agcggcgcga**tgcgaaagcacccgcaaaccgc (*Bam*HI)		Cloning of pyrG in pET-28a (N-terminal TEV cleavage site plus SGA spacer)
**MB297**	TTTACAAGCTTcagccacgagacgcaggttcc (*Bam*HI)		
**MB298**	CTGCACGgGTCGCTGGTGCCCTACCTGGCGCC		Generation of the *pyrG* mutant coding for Val186 (instead of Gly)
**MB299**	CCAGCGACcCGTGCAGAAAAAACACGTCCTCC		

Table S1, related to Table 2. Profile of resistance of *M. tuberculosis* strain overexpressing *ethA*.

	MIC (µg/ml) in *M. tuberculosis* strains		
	H37Rv		82.14 mutant
	7904688	7947882	7947882
**pSODIT-2**	1	0.5	> 10
**pSODIT/*ethA***	0.25-0.5	< 0.06	0.125

Table S2, related to Figure 1. MIC values of *M. tuberculosis ethA* and *pyrG* mutants to 11426026.

		MIC (µg/ml) in *M. tuberculosis*		
**Compounds**	**Structure**	**H37Rv**	***ethA* mutant (81.10)**	***pyrG* mutant (88.7)**
**11426026**	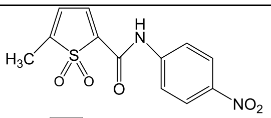	1	1	2.5
**7947882**	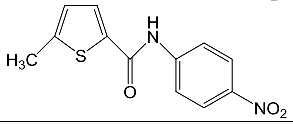	0.5	>40	5-10

Table S3, related to Figure 3. **Crystallographic data collection and refinement statistics. Values relative to the highest resolution shell are within parentheses.**

	**PyrG apo enzyme**	**PyrG in complex with two molecules of UTP**	**PyrG in complex with UTP/L-DON/AMP-PCP**
**Data collection**			
Wavelength (Å)	0.9801	0.9801	0.9150
Space group	$P4_3$	I222	$I2_12_12_1$
Cell dimensions *a*, *b*, *c* (Å)	196.83, 196.83, 184.04	79.41, 132.73, 157.64	121.73, 194.72, 207.46
α, β, γ (°)	90.0, 90.0, 90.0	90.0, 90.0, 90.0	90.0, 90.0, 90.0
Resolution (Å)	49.21 – 3.52 (3.58 – 3.52)	48.86 – 1.99 (2.04 – 1.99)	48.68 – 3.49 (3.67 – 3.49)
R_{merge}	0.137 (0.998)	0.052 (0.717)	0.091 (1.128)
$I/\sigma(I)$	8.6 (1.3)	13.5 (1.7)	16.7 (2.1)
CC(1/2) (%)	99.4 (61.3)	99.9 (66.6)	99.9 (76.6)
Unique reflections	85758	56696	31727
Completeness (%)	98.4 (92.1)	99.2 (92.9)	99.6 (98.0)
Redundancy	3.8 (3.7)	3.7 (3.6)	8.8 (8.6)
**Refinement**			
Resolution (Å)	3.52	1.99	3.49
No. reflections*	81332	53800	30084
R_{work}/R_{free} (%)	21.3/22.1	16.7/18.9	20.3/21.3
Average B-factors ($Å^2$)			
Macromolecule	111.00	40.62	143.57
Solvent	-	45.88	-
R.m.s deviations			
Bond lengths (Å)	0.008	0.010	0.008
Bond angles (°)	0.89	0.97	1.01
**PDB #**	4ZDI	4ZDJ	4ZDK

* Excluding the R_{free} set (5% of total reflections)

Table S4 related to Figure 5. MIC values of 7947882 derivatives. (A) Derivatives in which the thiophene moiety was substituted with a furan, a pyrazole, or a methyl thiazole moiety. (B) Derivatives in which the nitroalinine moiety was substituted. (C) Derivatives lacking the methyl in position 5 of the thiophene moiety. (D) Derivatives with a methylation in position 4 of the thiophene moiety. (E) Derivatives carrying a nitro group in position 4 of the thiophene moiety. (F) Carboxyl derivatives. (Excel File)

Table S5, related to Figure 5. Biological and biochemical characterization of 11426026 derivatives.					
**compound**	**structure**	**H37Rv MIC (µg/ml)**	**Ki (mM) PyrG wild type**	**[a]% of inhibition at 200 µM PyrG wild-type**	**[a]% of inhibition at 200 µM PyrG V186G mutant**
**11426026**	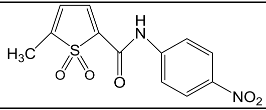	1	0.010 ± 0.002	83.6 ± 3.8	18.3 ± 1.5
**11426169**	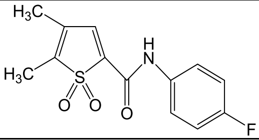	2.5	0.016 ± 0.004	84.8 ± 2.9	20.6 ± 1.2
**11426170**	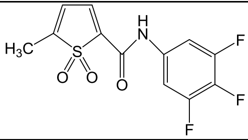	>20	0.032 ± 0.003	80.5 ± 1.7	9.5 ± 3.5
**11426171**	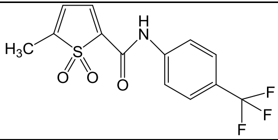	10	not determined	45.0 ± 3.2	4.3 ± 1.9
**11426172**	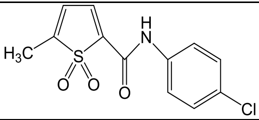	2.5	0.023 ± 0.004	83.4 ± 2.0	13.5 ± 1.9
**11426173**	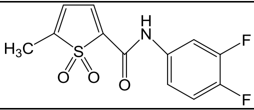	>5	0.044 ± 0.003	77.3 ± 2.1	14.1 ± 2.8
**11426174**	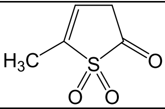	20	not inhibitory	not inhibitory	not inhibitory

[a]determined at a concentration of ATP equal to the K_m value (0.2 mM and 1.5 mM for the wild type and the mutant, respectively)

SUPPLEMENTAL EXPERIMENTAL PROCEDURES

Bacterial strains and growth conditions.

Cloning steps were performed in *Escherichia coli* XL1-Blue, following standard methods (Sambrook and Russel, 2001). For expression studies, the *E. coli* strains utilized were: One Shot® Mach1™-T1R (Invitrogen), and BL21(DE3).

E. coli cultures were grown either in Luria-Bertani (LB) broth or on LB agar. When necessary, antibiotics were added at the following concentrations: ampicillin, 100 µg/ml; hygromicin, 200µg/ml (20 µg/ml for *M. tuberculosis*); and kanamycin, 50 µg/ml.

Both 7947882 [5-methyl-N-(4-nitrophenyl)-2-thiophenecarboxamide] and 7904688 [3-phenyl-N-({[4-(1-piperidinyl)phenyl]-amino} carbonothioyl) propanamide] compounds were dissolved in dimethyl sulfoxide (DMSO) (http://www.chembridge.com/index.php).

M. tuberculosis strains were grown aerobically at 37°C either in Middlebrook 7H9 medium or on Middlebrook 7H11 agar, both supplemented with 10% OADC Middlebrook Enrichment.

Library screening

Compounds from a selected library from NIAID were initially screened at 10 µg/ml in duplicate for activity against H37Rv and ss18b model in 96-well format using the resazurin reduction assay (REMA). Briefly, compounds (1 µl of 10mg/ml in DMSO) were added to the wells of a 96 well plate. Column 1 served as a negative control (DMSO), and column 12 served as a positive control (final concentration of rifampicin:0.1 µg/ml for H37Rv or 2 µg/ml for ss18b). For the H37Rv plates, frozen aliquots diluted into Middlebrook 7H9 (supplemented with 10% ADC, 0.2% glycerol, 0.05% tween 80) to an $OD_{600\ nm}$ of 0.0001 (100 µl) were added to each well. For the ss18b plates, frozen aliquots of ss18b (Sala et al., 2010) diluted into 7H9 to an $OD_{600\ nm}$ of 0. 1 (100 µl) were added to each well. Following 1 week incubation (37°C), bacterial viability was determined using the resazurine reduction assay (add 10 µl of 0.025% resazurin). Following an overnight incubation,

the fluorescence of the resazurin metabolite resorufin was determined (excitation at 560 nm and emission at 590 nm; gain, 80) by using a Tecan Infinite M200 microplate reader.

The cytotoxicity of the compounds was determined on human hepatocellular carcinoma cell line HepG2 and Huh7, human lung epithelial cell line; A549 and murine macrophagecell line; RAW 264.7. Briefly, serial dilutions of the compounds were added to the well of a 96 well plate (1 in 2 dilutions, from 40 to 0.08 µg/ml final concentration), column 1 served as negative controland column 12 as positive control (no cells added). Subsequently, 100 µl of the cell lines was seeded into the 96 well plates (5 x 10^3 cells per well, in DMEM containing 10% Fetal Bovine Serum (HepG2, Huh7 and A549), or RPMI containing 10% Fetal Bovine Serum (Raw264.7). Plates were incubated for 3 days (37°C with 5% CO_2) and viability determined using resazurin. Plates were incubated for 2 more hours and cell viability was determined by fluorescence (excitation 570 nm, emission 590 nm).

MIC determinations.

A single colony of each *M. tuberculosis* strain was inoculated in complete Middlebrook 7H9. Cell cultures were grown at 37°C until exponential growth phase (~10^8CFU/ml) was reached.

Dilutions to the final concentration of ~10^6CFU/ml were performed and about 1 µl of cell culture was streaked onto plates containing two-fold serial dilutions of appropriate compound. MIC values were assigned as the lowest drug concentrations inhibiting bacterial growth. All experiments were repeated three times.

Infection of THP-1-derived macrophages.

THP-1 monocytes (American Type Culture Collection) were grown in suspension at 37°C in 5% CO_2 in bicarbonate-buffered RPMI (Gibco) supplemented with 10% (vol/vol) fetal bovine serum (FBS; Gibco), 50 µmol/liter β-mercaptoethanol to a density of about 0.5x10^6 cells/ml. Differentiation of monocytes into macrophages was obtained by plating the cells in 96-well plates at

a density of about 7.5x10^4 cells/well in the presence of 50 ng/ml phorbol 12-myristate 13-acetate (PMA; Sigma-Aldrich). After 24 h, PMA was removed and cells were infected with *M. tuberculosis* pyrG conditional mutant and its control strain at a multiplicity of infection of 1:20 (number of CFU/cell) for 90 min (Manganelli et al., 2001). After infection, extracellular bacteria were removed by washing with phosphate-buffered saline (PBS) and fresh RPMI medium with or without ATc (200 ng/ml). The medium was replaced every 48 h. At different time points, macrophages were lysed with SDS (0.05%). The lysate containing the bacteria was diluted in fresh medium and plated to determine the viable counts (Manganelli et al., 2001).

Expression and purification of *M. tuberculosis* EthA.

The *ethA* gene from *M .tuberculosis* H37Rv was amplified by standard PCR. The PCR fragment was cloned in the T7 promoter-based expression vector pET-SUMO, generating pET-SUMO/*ethA*. One or more *E. coli* fresh colonies carrying pET SUMO/*ethA* recombinant plasmid were used to transform *E.coli* BL21(DE3) One Shot® cells, that were plated onto LB agar plates containing 50 µg/ml kanamycin. For each transformation, a single colony overnight pre-inoculum, grown at 37°C in the presence of 50 µg/ml kanamycin, was diluted 1:50 in 6 liters of the same medium. The culture was then incubated at 37°C until OD_{600nm} 0.6-0.8 was reached; therefore, isopropyl-β-thiogalactopyranoside (IPTG) was added at final concentration of 0.5mM, and the incubation was continued for 3h. Cells were harvested by centrifugation, resuspended in 50 mM sodium phosphate pH 8.0, 300 mM NaCl, 1% triton-X100, and disrupted by sonication. The cell-free extract was obtained by centrifuging at 30,000g for 45 minutes at 4°C and applied onto a nickel affinity column (1 ml, Ni-NTA, Qiagen), washed with 100 mM imidazole and the protein eluted with 250 mM imidazole. Tag cleavage was achieved by overnight incubation with 0.3 mg of SUMO protease dialyzed against 50 mM sodium phosphate pH 8.0, followed by a second Ni-NTA purification step to remove both tag and SUMO protease. Samples purity was checked by SDS-PAGE and proteins concentration evaluated by absorbance at 280 nm (ε=97290 M^{-1} cm^{-1}).

Expression and purification of *M. tuberculosis* PyrG.

The *PyrG* gene from *M .tuberculosis* H37Rv was amplified by standard PCR. The PCR fragment was cloned in the pET28a plasmid, generating pET28a/*PyrG*. *M. tuberculosis* PyrG wild type recombinant protein was obtained fused with a His_6 tag in *E. coli* BL21(DE3) cells grown in LB medium containing 50 µg/ml kanamycin at 25°C for 12 hours, after induction with 0.5 mM IPTG. For all enzymatic assays, the protein was purified through a nickel affinity column in 50 mM sodium phosphate pH 8.0, 300 mM NaCl, washed with 25 mM imidazole, eluted with 250 mM imidazole in the same buffer and further dialyzed in 50 mM potassium phosphate pH 7.5, 50 mM KCl. For structural studies, PyrG was similarly purified through a first IMAC step on a HisTrap column (GE Healthcare) in 50 mM Tris-HCl pH 8.0, 500 mM NaCl, 25 mM imidazole, 5% glycerol and eluted applying a 25 to 400 mM imidazole gradient in the same buffer. The purified enzyme was then dialyzed in 25 mM Hepes pH 8.0, 150 mM NaCl, 5% glycerol, 1 mM DTT after adding recombinant TEV (Van Den Berg et al., 2006) to a 1:30 w/w ratio. The dialyzed sample was further purified from the protease and the cleaved tag by a second, gravity flow step through 1.0 ml of Ni-NTA resin (Qiagen), concentrated and injected onto a HiLoad 26/60 Superdex 200 size exclusion column (GE Healthcare), equilibrated in 25 mM Hepes-Na pH 8.0, 150 mM NaCl, 5% glycerol and run at 1 ml/min.

Samples purity was checked by SDS-PAGE and proteins concentration evaluated by absorbance at 280 nm (ε=40715 M^{-1} cm^{-1}).

The PyrG resistant mutant (V186G) protein was obtained by site-directed mutagenesis on the corresponding plasmid, using the QuikChange procedure (Agilent) and primers designed to include the desired mutation. The mutant enzyme was expressed and purified as described for the wild-type (see above).

Enzymatic activity assays, steady state kinetics and inhibition assays.

EthA enzyme activity towards compounds was determined spectrophotometrically by monitoring the decrease of NADPH at 340 nm (ε= 6.22 mM^{-1} cm^{-1}) at 37°C as previously reported (Fraaije et al, 2004), using an Eppendorf BioSpectrometer. Reaction mixtures typically contained 50 mM potassium phosphate pH 8.0, 0.2 mM NADPH, 10 μM serum bovine albumin (BSA), 50 μM of compound (dissolved in dimethylformamide), and the reaction was started by adding the enzyme solution (1 μM).

PyrG activity was determined at 37°C using a continuous spectrophotometric assay following the rate of increase in absorbance at 291 nm due to the conversion of UTP to CTP (ε= 1.34 mM^{-1} cm^{-1}) (Lunn et al., 2008). Assays were performed at 37°C in 50 mM HEPES pH 8.0, 10 mM $MgCl_2$, 1 mM UTP, 1 mM ATP, 0.5 μM PyrG, and the reaction was started by the addition of 100 mM NH_4Cl.

Steady-state kinetics parameters were determined by assaying the enzymes at least at 8 different concentrations of their substrates. All experiments were performed in triplicate, and the kinetic constants, K_m and k_{cat}, were determined fitting the data to the Michaelis-Menten equation using Origin 8 software.

PyrG inhibition was initially screened for all compounds at 200 µM (dissolved in DMSO). For compounds that significantly inhibited the enzyme activity in these conditions, IC_{50} and K_i values were determined. For IC_{50} determinations, the enzyme activities were measured in presence of a serial dilution of each compound and values were estimated by fitting log[I] and normalized in response to Equation 1. The K_i values were determined using an adapted equation for competitive inhibition (Equation 2) (Copeland, 2000).

$$V = \frac{100}{\{1+10^{(\log(IC_{50}-[I])\times h)}\}} \qquad \text{equation 1}$$

$$v = \frac{V_{\max}[S]}{[S] + K_m\left(1 + \frac{[I]}{K_i}\right)} \qquad \text{equation 2}$$

***In vitro* EthA metabolites production and identification.**

For *in vitro* EthA metabolite production, 30 mg of 7947882 were incubated with 10 mg of EthA in 50 mM potassium phosphate pH 8.0, 500 μM NADPH, 10 μM BSA, at 37°C, 5 h under agitation. The reaction mixture was then partitioned between water and diethyl ether, the aqueous layer was extracted with diethyl ether, and combined organic layer washed with brine and dried over Na_2SO_4. Solvent was removed under reduced pressure and the residue was purified by flash column chromatography (Merck SiO_2 60, 230–400 mesh). Elution was performed with hexane-ethyl acetate ration 8:2. Visualization of reaction components was achieved under UV light at a wavelength of 254 nm, or staining by exposure to a 0.5% solution of vanillin in H_2SO_4/EtOH. Mass spectra were recorded in negative ESI resolution mode with a Thermo LTQ-XL mass spectrometer.

***In vitro* production of the PyrG-7947882 metabolite complex.**

In order to obtain the PyrG complexed with the EthA activated metabolite of 7947882 or of 7904688, the enzyme was incubated with each compound in the presence of the monooxygenase. Briefly, PyrG (45 μM) was incubated with EthA (10 μM) in 50 mM potassium phosphate buffer pH 8.0, 300 μM NADPH, 300 μM 7947882 or 7904688, at 37°C. For the blank control NADPH was omitted from the reaction mixture, in order to avoid prodrug activation. At regular intervals, aliquots were withdrawn and PyrG activity was measured, to determine the enzyme inhibition. The activity measurements were performed as described, but with a final concentration of ATP of 0.2 mM.

After 4 hours of incubation, the reaction mixture was loaded on a Ni-NTA column equilibrated in 50 mM potassium phosphate pH 7.5, 50 mM KCl and washed with the same buffer to elute EthA, unbound 7947882 (or 7904688) and metabolite(s). PyrG was then eluted with 100 mM imidazole in

the same buffer, dialyzed against 25 mM potassium phosphate pH 7.5, 50 mM KCl and concentrated.

Metabolomics Experiments

For liquid chromatography-mass spectrometry analysis of polar metabolites, the samples were diluted1:1 with acetonitrile containing 0.2% acetic acid. After centrifugation at 13000g for 10 minutes the samples were applied on a Cogent Diamond Hydride Type C silica column (150 mm × 2.1 mm; dead volume 315 µl) usingan Agilent 1200 LC system. The flow rate was 0.4 ml/min.The gradient is based on the number 3 (Pesek et al., 2008). An Agilent Accurate Mass 6230 TOF apparatus was employed. Dynamic mass axis calibration was achieved by continuous infusion of a reference mass solution using an isocratic pump connected to a multimode ionization source, operated in the positive-ion and negative-ion mode. ESI capillary and fragmentor voltages were set at 3500 V and 100 V, respectively. The nebulizer pressure was set at 40 psi and the nitrogen drying gas flow rate was set at 10 l/min. The drying gas temperature was maintained at 250°C. The MS acquisition rate was 1.5 spectra/sec and m/z data ranging from 50-1200 were stored. Data were collected in the centroid mode in the 4 GHz (extended dynamic range) mode. Detected m/z were deemed to identify metabolites on the basis of unique accurate mass-retention time identifiers for masses, exhibiting the expected distribution of accompanying isotopomers. Data were analyzed with the Qualitative Analysis software v. B.04.00. Heat map was generated using Heatmap Builder® V. 11 (King et al., 2005).

For TLC analysis of radiolabeled nucleotide extract, 1000-3000 dpm were removed from the formic acid extract, which was evaporated under vacuum. The sample was re-dissolved in 5 µl of water and loaded on PEI Cellulose F plate (Millipore). After drying of the sample, the TLC plate was soaked for 20 min in methanol, dried on air and developed in 0.75 M KH_2PO_4, pH adjusted to 3.5 with 0.75 M H_3PO_4 (Bochner and Ames, 1982). The radioactive spots were visualized by exposing the TLC

plate to BioMax MR autoradiography film at -80°C. 10 nmols of cold standards (UTP, UDP, UMP, CTP, CDP, CMP, UDP-Gal, UDP-GlcNAc; Sigma-Aldrich) were loaded on the TLC plate and located by UV (λ=254 nm).

For HPLC analysis of radiolabeled nucleotide extract, aliquots of formic acid extracts corresponding to 4000 dpm (GAS) or 7000 dpm (7H9) were evaporated under vacuum. These samples were then re-dissolved in 20 µl of water and combined with 3 µl of the cold standards mixture [UTP, UDP, UMP, CTP, CDP, CMP, UDP-Gal, UDP-GlcNAc, UDP-MurNAc-pentapeptide (the latter from BaCWAN Synthetic Facility, University of Warwick)]. 20 µl of this sample was separated on BioBasic AX anion exchange HPLC column (Singh et al., 2015). Radioactivity was determined by scintillation spectrometry using 5 ml of EcoLite scintillation liquid.

Docking and cheminformatics.

The protein was prepared for docking using the default settings of the 'prepare protein' protocol in Discovery Study 4.1 (Biovia, San Diego, CA). The protein was used for docking using LibDock (Rao et al., 2007). The proposed binding sites (ATP and UTP) were centered on the UTP molecules. The coordinates for the ATP site was X = 8.71, Y = 24.72, Y = 66.82 (sphere radius 9.19A), while for the UTP site was X = 11.65, Y = 65.49, Z = 8.54, (sphere radius 8.54A). The protocol included 100 hotspots and docking tolerance (0.25). The FAST conformation method was also used along with steepest descent minimization with CHARMm. The 11426026 structure was initially docked in both sites.

A substructure of 11426026 (4-Nitroacetanilide) was used to search for a set of *M. tuberculosis* active compounds (Ananthan et al., 2009; Ekins et al., 2014; Maddry et al., 2009; Reynolds et al., 2012) collected in the Collaborative Drug Discovery Database (www.collaborativedrug.com, Burlingame, CA; Ekins and Bunin, 2013). Twelve compounds were retrieved and docked in the

PyrG ATP site. Molecules were visualized, and their 2D interaction plots generated and selected for follow-up.

Synthesis of thiophene derivatives.

Visualization of reaction components was achieved under UV light at a wavelength of 254 nm. ^{1}H and ^{13}C NMR spectra were recorded in Bruker AC-300. Chemical shifts are given in ppm and are referenced by using the residual signals of the solvent as internal standard. Mass spectra were recorded with a Waters ZQ-2000 mass spectrometer.

5-Acetylthiophene-2-carboxylic acid (11326027), 5-methylthiophene-2-carboxylic acid (11326028), 5-methyl-4-nitrothiophene-2-carboxylic acid (11326034), 5-methylthiophene-2-carboxamide (11226085), thiophene-2-carboxylic acid (11326226), 5-methyl-2-furoic acid (11326227), 3-methylthiophene-2-carboxylic acid (11326228), 4-methyl-1,3-thiazole-5-carboxylic acid (11326229), 4-methylthiophene-2-carboxylic acid (11326230), 1-ethyl-3-methyl-1*H*-pyrazole-5-carboxylic acid (11326231) were purchased from Sigma-Aldrich Co (USA) and used after one recrystallisation from suitable solvent.

Synthesis of thiophene derivatives- Solution of thiophene-2-carboxilic acid in CCl_4 was treated thionyl chloride and one drop of DMF was added. The reaction mixture was refluxed for 2 hours and evaporated under vacuum and residue was used in the next step without additional purification. Solution of chlor anhydride from previous step in CH_3CN was treated by 2 mol of corresponding amine at room tempetarure, reaction mixture was stored for 4 hours, diluted by cold water and precipitate was filtered off. Thiophene-2-carboxamide was purified by the crystallization from ethanol.

Water hydrogen peroxide was added by drop to trifluoroacetic acid and stored for 1 hour at room temperature. Thiophene-2-carboxamide was added to this mixture, it was heated to 70 ^{o}C for 24 hours, diluted by cold water and precipitate was filtered off. Thiophene-2-carboxamide 1,1-dioxide was purified by the crystallization from ethanol.

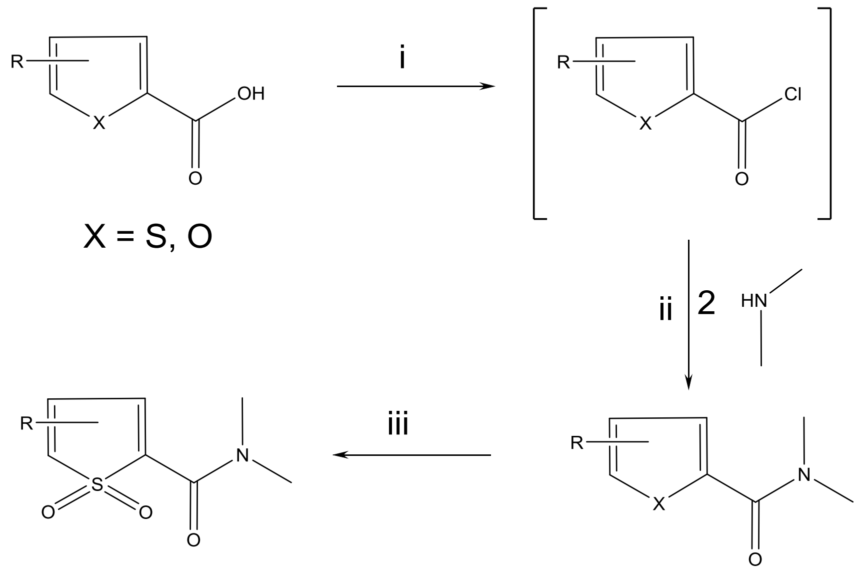

Scheme 1. Reagents and conditions: (i) $SOCl_2$, DMF, CCl_4, reflux, 2 h, 100%; (ii) CH_3CN, 20 °C, 4 h; (iii) H_2O_2 H_2O, CF_3COOH, 70 °C.

Analytical data of thiophene derivatives

5-Acetyl-*N*-(4-nitrophenyl)thiophene-2-carboxamide (11226089): Yield 42%, M.p. 143-6 °C, ^{1}H NMR (300 MHz, DMSO-d6): 9.39 (br s, 1H), 8.22 (d, 2H, *J* = 9.6 Hz), 7.77 (d, 2H, *J* = 9.6 Hz), 7.44 (d, 1H, *J* = 3.7 Hz), 6.86 (d, 1H, *J* = 3.7 Hz), 2.74 (s, 3H); LCMS (ESI): *m/z* 291.3 $(M+H)^+$.

***N*-(3,5-Dichlorobenzyl)-*N*,5-dimethylthiophene-2-carboxamide (11326001):** Yield 69%, M.p. 73-5 °C, ^{1}H NMR (300 MHz, DMSO-d6): 7.41 (s, 1H), 7.29 (d, 1H, *J* = 3.7 Hz), 7.01 (d, 1H, *J* = 3.7 Hz), 4.45 (s, 2H), 2.93 (s, 3H), 2.44 (s, 3H); LCMS (ESI): *m/z* 315.2 $(M+H)^+$.

***N*-(2,3-Difluorophenyl)-5-methylthiophene-2-carboxamide (11326002):** Yield 62%, M.p. 105-07 °C, ^{1}H NMR (300 MHz, DMSO-d6): 9.73 (br s, 1H), 7.59 (m, 1H), 7.39 (d, 1H, *J* = 3.7 Hz), 6.99 (d, 1H, *J* = 3.7 Hz), 6.58 (m, 2H), 2.43 (s, 3H); LCMS (ESI): *m/z* 254,3 $(M+H)^+$.

***N*-(2-Fluorophenyl)-5-methylthiophene-2-carboxamide (11326003):** Yield 66%, M.p. 130-32 °C, ^{1}H NMR (300 MHz, DMSO-d6): 9.47 (br s, 1H), 7.89 (m, 1H), 7.77 (m, 1H), 7.42 (d, 1H, *J* = 3.7 Hz), 7.06 (m, 1H), 6.98 (d, 1H, *J* = 3.7 Hz), 6.91 (m, 1H), 2.44 (s, 3H); LCMS (ESI): *m/z* 236.3 $(M+H)^+$.

5-Methyl-*N*-(3,4,5-trifluorophenyl)thiophene-2-carboxamide (11326005): Yield 69%, M.p. 152-54 °C, ^{1}H NMR (300 MHz, DMSO-d6): 9.52 (br s, 1H), 7.49 (m, 2H), 7.41 (d, 1H, *J* = 3.7 Hz), 6.91 (d, 1H, *J* = 3.7 Hz), 2.41 (s, 3H); LCMS (ESI): *m/z* 272.3 $(M+H)^+$.

***N*-(4-Fluorophenyl)-5-methylthiophene-2-carboxamide (11326006):** Yield 72%, M.p. 140-42 ^{o}C, ^{1}H NMR (300 MHz, DMSO-d6): 9.20 (br s, 1H), 7.66 (m, 2H), 7.44 (d, 1H, *J* = 3.7 Hz), 6.99 (m, 2H), 6.87 (d, 1H, *J* = 3.7 Hz), 2.44 (s, 3H); LCMS (ESI): *m/z* 236.3 $(M+H)^{+}$.

5-Methyl-*N*-[4-(trifluoromethoxy)phenyl]thiophene-2-carboxamide (11326007): Yield 76%, M.p. 170-72 ^{o}C, ^{1}H NMR (300 MHz, DMSO-d6): 9.18 (br s, 1H), 7.83 (d, 2H, *J* = 6.9 Hz), 7.47 (d, 1H, *J* = 3.7 Hz), 7.29 (d, 2H, *J* = 6.9 Hz), 6.79 (d, 1H, *J* = 3.7 Hz), 2.40 (s, 3H); LCMS (ESI): *m/z* 302.3 $(M+H)^{+}$.

***N*-(3,4-Dimethoxyphenyl)-5-methylthiophene-2-carboxamide (11326008):** Yield 59%, M.p. 146-48 ^{o}C, ^{1}H NMR (300 MHz, DMSO-d6): 10.21 (br s, 1H), 7.48 (d, 1H, *J* = 3.7 Hz), 7.43 (s, 3H), 7.10 (d, 2H, *J* = 9.2 Hz),), 6.83 (d, 1H, *J* = 9.2 Hz), 6.74 (d, 2H, *J* = 3.7 Hz), 4.01 (s, 3H), 3.67 (s, 3H), 2.45 (s, 3H); LCMS (ESI): *m/z* 278.3 $(M+H)^{+}$.

***N*,5-Dimethyl-*N*-(4-methylphenyl)thiophene-2-carboxamide (11326009):** Yield 59%, M.p. 146-48 ^{o}C, ^{1}H NMR (300 MHz, DMSO-d6): 7.40 (d, 1H, *J* = 3.7 Hz), 7.19 (m, 4H), 6.76 (d, 2H, *J* = 3.7 Hz), 3.65 (s, 3H), 2.44 (s, 3H), 2.35 (s, 3H); LCMS (ESI): *m/z* 246.3 $(M+H)^{+}$.

***N*-[4-(Aminosulfonyl)phenyl]-5-methylthiophene-2-carboxamide (11326010):** Yield 69%, M.p. 80-2 ^{o}C, ^{1}H NMR (300 MHz, DMSO-d6): 9.64 (br s, 1H), 7.86 (br s, 2H), 7.82 (d, 2H, *J* = 7.2 Hz), 7.58 (d, 2H, *J* = 7.2 Hz), 7.47 (d, 1H, *J* = 3.7 Hz), 6.79 (d, 1H, *J* = 3.7 Hz), 2.42 (s, 3H); LCMS (ESI): *m/z* 297.4 $(M+H)^{+}$.

***N*-{2-[4-(Aminosulfonyl)phenyl]ethyl}-5-methylthiophene-2-carboxamide (11326011):** Yield 54%, M.p. 212-14 ^{o}C, ^{1}H NMR (300 MHz, DMSO-d6): 7.81 (d, 2H, *J* = 7.2 Hz), 7.47 (d, 1H, *J* = 3.7 Hz), 7.30 (d, 2H, *J* = 7.2 Hz), 6.85 (d, 1H, *J* = 3.7 Hz), 6.10 (br s, 2H), 3.45 (t, 2H, *J* = 6.5 Hz), 2.87 (t, 2H, *J* = 6.5 Hz), 2.44 (s, 3H); LCMS (ESI): *m/z* 325.4 $(M+H)^{+}$.

***N*-(3-Chlorobenzyl)-5-methylthiophene-2-carboxamide (11326012):** Yield 58%, M.p. 105-07 ^{o}C, ^{1}H NMR (300 MHz, DMSO-d6): 7.79 (s, 1H), 7.36 (d, 1H, *J* = 3.7 Hz), 7.26 (m, 2H), 7.18 (m, 1H), 7.10 (s, 1H), 7.08 (s, 1H), 6.91 (d, 1H, *J* = 3.7 Hz), 4.41 (d, 2H, *J* = 1.1 Hz), 2.42 (s, 3H); LCMS (ESI): *m/z* 266.8 $(M+H)^{+}$.

***N*-[2-(4-Ethoxyphenyl)ethyl]-5-methylthiophene-2-carboxamide (11326014):** Yield 65%, M.p. 118-20 ^{o}C, ^{1}H NMR (300 MHz, DMSO-d6): 7.83 (br s, 1H), 7.47 (d, 1H, *J* = 3.7 Hz), 7.15 (d, 2H, *J* = 8.7 Hz), 6.88 (d, 1H, *J* = 3.7 Hz), 6.84 (d, 2H, *J* = 8.7 Hz), 4.51 (q, 2H, *J* = 7.4 Hz), 3.43 (t, 2H, *J* = 6.4 Hz), 2,74 (t, 2H, *J* = 6.6 Hz), 2.44 (s, 3H), 1.44 (t, 3H, d, *J* = 7.4 Hz); LCMS (ESI): *m/z* 290.4 $(M+H)^{+}$.

***N*-(2,2-Diphenylethyl)-5-methylthiophene-2-carboxamide (11326015):** Yield 54%, M.p. 144-46 ^{o}C, ^{1}H NMR (300 MHz, DMSO-d6): 7.45 (d, 1H, *J* = 3.7 Hz), 7.28 (m, 4H), 7.23 (m, 6H), 6.98 (br

s, 1H), 6.83 (d, 1H, *J* = 3.7 Hz), 4.79 (t, 1H, *J* = 7.1 Hz), 4.05 (d, 2H, *J* = 7.1 Hz), 2.41 (s, 3H); LCMS (ESI): *m/z* 322.4 $(M+H)^+$.

1-(Diphenylmethyl)-4-[(5-methyl-2-thienyl)carbonyl]piperazine (11326016): Yield 57%, M.p. 88-90 ^{o}C, ^{1}H NMR (300 MHz, DMSO-d6): 7.51 (d, 1H, *J* = 3.7 Hz), 7.29 (m, 4H), 7.21 (m, 6H), 6.83 (d, 1H, *J* = 3.7 Hz), 4.60 (s, 1H), 3.72 (m, 4H), 3.04 (m, 4H), 2.46 (s, 3H); LCMS (ESI): *m/z* 377.5 $(M+H)^+$.

***N*-[3-(Aminosulfonyl)phenyl]-5-methylthiophene-2-carboxamide (11326017):** Yield 32%, M.p. 240-42 ^{o}C, ^{1}H NMR (300 MHz, DMSO-d6): 9.45 (br s, 1H), 8.02 (m, 1H), 7.74 (br s, 2H), 7.59 (m, 1H), 7.53 (m, 1H), 7.50 (d, 1H, *J* = 3.7 Hz), 7.18 (m, 1H), 6.71 (d, 1H, *J* = 3.7 Hz), 2.42 (s, 3H); LCMS (ESI): *m/z* 297.4 $(M+H)^+$.

***N*-(2,2-Difluoro-1,3-benzodioxol-5-yl)-5-methylthiophene-2-carboxamide (11326018):** Yield 75%, M.p. 136-38 ^{o}C, ^{1}H NMR (300 MHz, DMSO-d6): 10.12 (br s, 1H), 7.76 (s, 1H), 7.43 (d, 1H, *J* = 3.7 Hz), 7.41 (d, 1H, *J* = 8.6 Hz), 7.15 (d, 1H, *J* = 8.6 Hz), 6.79 (d, 1H, *J* = 3.7 Hz), 2.40 (s, 3H); LCMS (ESI): *m/z* 298.3 $(M+H)^+$.

1-[(5-Methyl-2-thienyl)carbonyl]-1,2,3,4-tetrahydroquinoline (11326019): Yield 53%, M.p. 104-06 ^{o}C, ^{1}H NMR (300 MHz, DMSO-d6): 7.85 (d, 1H, *J* = 7.1 Hz), 7.38 (d, 1H, *J* = 3.7 Hz), 7.24 (t, 1H, *J* = 8.0 Hz), 7.12 (t, 1H, *J* = 8.0 Hz), 6.89 (t, 1H, *J* = 7.1 Hz), 6.81 (d, 1H, *J* = 3.7 Hz), 3.94 (m, 2H), 3.27 (m, 2H), 2.49 (m, 2H), 2.39 (s, 3H); LCMS (ESI): *m/z* 258.4 $(M+H)^+$.

***N*-2-Adamantyl-5-methylthiophene-2-carboxamide (11326020):** Yield 58%, M.p. 152-54 ^{o}C, ^{1}H NMR (300 MHz, DMSO-d6): 7.59 (d, 1H, *J* = 3.7 Hz), 7.01 (t, 1H, *J* = 3.7 Hz), 6.71 (br s, 1H), 3.98 (m, 1H), 2.48 (s, 3H), 1.93-1.42 (m, 14H); LCMS (ESI): *m/z* 276.4 $(M+H)^+$.

5-Methyl-*N*-[4-(trifluoromethyl)phenyl]thiophene-2-carboxamide (11326021): Yield 63%, M.p. 188-90 ^{o}C, ^{1}H NMR (300 MHz, DMSO-d6): 9.16 (br s, 1H), 7.77 (m, 2H), 7.68 (m, 2H), 7.48 (d, 1H, *J* = 3.7 Hz), 6.92 (d, 1H, *J* = 3.7 Hz), 2.35 (s, 3H); LCMS (ESI): *m/z* 286.3 $(M+H)^+$.

***N*-[2-(2,5-Dimethoxyphenyl)ethyl]-5-methylthiophene-2-carboxamide (11326022):** Yield 60%, M.p. 68-70 ^{o}C, ^{1}H NMR (300 MHz, DMSO-d6): 7.48 (d, 1H, *J* = 3.7 Hz), 6.91 (br s, 1H), 6.86 (d, 1H, *J* = 3.7 Hz), 6.81 (s, 1H), 6.74 (d, 1H, *J* = 8.6 Hz), 6.61 (d, 1H, *J* = 8.6 Hz), 3.75 (s, 3H), 3.67 (s, 3H), 3.50 (t, 2H, *J* = 7.2 Hz), 2.74 (t, 2H, *J* = 7.2 Hz), 2.49 (s, 3H); LCMS (ESI): *m/z* 306.4 $(M+H)^+$.

***N*-(3-Bromo-4-methoxyphenyl)-5-methylthiophene-2-carboxamide (11326023):** Yield 66%, M.p. 134-6 ^{o}C, ^{1}H NMR (300 MHz, DMSO-d6): 8.96 (br s, 1H), 7.78 (s, 1H), 7.49 (d, 1H, *J* = 3.7 Hz), 7.41 (d, 1H, *J* = 8.7 Hz), 6.91 (d, 1H, *J* = 8.7 Hz), 6.78 (d, 1H, *J* = 3.7 Hz), 3.84 (s, 3H), 2.44 (s, 3H); LCMS (ESI): *m/z* 327.2 $(M+H)^+$.

***N*-1-Adamantyl-5-methylthiophene-2-carboxamide (11326024):** Yield 76%, M.p. 164-6 ^{o}C, ^{1}H NMR (300 MHz, DMSO-d6): 7.51 (d, 1H, *J* = 3.7 Hz), 6.94 (d, 1H, *J* = 3.7 Hz), 4.66 (br s, 1H), 2.53 (s, 3H), 2.01 (br s, 3H), 1.67 (m, 12H); LCMS (ESI): *m/z* 276.4 $(M+H)^{+}$.

***N*-Ethyl-5-methyl-*N*-phenylthiophene-2-carboxamide (11326025):** Yield 72%, M.p. 68-70 ^{o}C, ^{1}H NMR (300 MHz, DMSO-d6): 7.62 (m, 2H), 7.43 (d, 1H, *J* = 3.7 Hz), 7.29 (m, 3H), 6.81 (d, 1H, *J* = 3.7 Hz), 3.86 (q, 2H, *J* = 7.0 Hz), 2.32 (s, 3H), 1.21 (t, 3H, *J* = 7.0 Hz); LCMS (ESI): *m/z* 246.3 $(M+H)^{+}$.

***N*-(4-Ethylphenyl)-5-methylthiophene-2-carboxamide (11326026):** Yield 57%, M.p. 128-30 ^{o}C, ^{1}H NMR (300 MHz, DMSO-d6): 9.26 (br s, 1H), 7.57 (d, 1H, *J* = 3.7 Hz), 7.42 (m, 2H), 7.35 (m, 3H), 6.70 (d, 1H, *J* = 3.7 Hz), 2.60 (q, 2H, *J* = 9.0 Hz), 2.32 (s, 3H), 1.18 (t, 3H, *J* = 9.0 Hz); LCMS (ESI): *m/z* 246.3 $(M+H)^{+}$.

***N*-[(1*Z*)-(Dimethylamino)methylene]-5-methylthiophene-2-carboxamide (11326033):** Yield 67%, M.p. 87-89^{0}C, ^{1}H NMR (300 MHz, DMSO-d6): 7.63 (br s, 1H), 7.54 (d, 1H, *J* = 3.7 Hz), 6.79 (d, 1H, *J* = 3.7 Hz), 3.14 (s, 6H), 2.41 (s, 3H); LCMS (ESI): *m/z* 197.3 $(M+H)^{+}$.

***N*-[(1*E*)-Anilinomethylene]-5-methylthiophene-2-carboxamide (11326035):** Yield 50%, M.p. 162-4 ^{o}C, ^{1}H NMR (300 MHz, DMSO-d6): 10.04 (br s, 1H), 7.68 (d, 1H, *J* = 3.7 Hz), 7.51 (m, 2H), 7.16 (m, 3H), 7.01 (s, 1H), 6.83 (d, 1H, *J* = 3.7 Hz), 2.51 (s, 3H); LCMS (ESI): *m/z* 245.3 $(M+H)^{+}$.

5-Methyl-*N*-{(1*E*)-[(4-nitrophenyl)amino]methylene}thiophene-2-carboxamide (11326036): Yield 59%, M.p. 115-7 ^{o}C, ^{1}H NMR (300 MHz, DMSO-d6): 10.66 (br s, 1H), 8.50 (d, 2H, *J* = 9.3 Hz), 7.74 (d, 2H, *J* = 9.3 Hz), 7.61 (d, 1H, *J* = 3.7 Hz), 7.13 (s, 1H), 6.84 (d, 1H, *J* = 3.7 Hz), 2.49 (s, 3H); LCMS (ESI): *m/z* 290.3 $(M+H)^{+}$.

***N*-{(1*E*)-[(2,3-Difluorophenyl)amino]methylene}-5-methylthiophene-2-carboxamide (11326037):** Yield 47%, M.p. 158-60 ^{o}C, ^{1}H NMR (300 MHz, DMSO-d6): 10.87 (br s, 1H), 7.69 (d, 1H, *J* = 3.7 Hz), 7.11 (m, 1H), 6.76 (d, 1H, *J* = 3.7 Hz), 6.69 (m, 1H), 6.59 (m, 1H), 2.51 (s, 3H); LCMS (ESI): *m/z* 281.3 $(M+H)^{+}$.

5-Methyl-*N*-((1*E*)-{[4-(trifluoromethoxy)phenyl]amino}methylene)thiophene-2-carboxamide (11326038): Yield 51%, M.p. 105-7 ^{o}C, ^{1}H NMR (300 MHz, DMSO-d6): 10.31 (br s, 1H), 7.76 (d, 2H, *J* = 9.0 Hz), 7.47 (d, 1H, *J* = 3.7 Hz), 7.35 (d, 2H, *J* = 9.0 Hz), 7.13 (s, 1H), 6.83 (d, 1H, *J* = 3.7 Hz), 2.32 (s, 3H); LCMS (ESI): *m/z* 329.3 $(M+H)^{+}$.

5-Methyl-*N*-{(1*E*)-[methyl(4-methylphenyl)amino]methylene}thiophene-2-carboxamide (11326039): Yield 56%, M.p. 82-84 ^{o}C, ^{1}H NMR (300 MHz, DMSO-d6): 7.78 (s, 1H), 7.53 (d, 1H, *J* = 3.7 Hz), 7.39 (d, 2H, *J* = 9.0 Hz), 7.31 (d, 2H, *J* = 9.0 Hz), 6.78 (d, 1H, *J* = 3.7 Hz), 3.34 (s, 3H), 2.53 (s, 3H), 2.29 (s, 3H); LCMS (ESI): *m/z* 273.4 $(M+H)^{+}$.

***N*-((1*E*)-{[2-(4-Ethoxyphenyl)ethyl]amino}methylene)-5-methylthiophene-2-carboxamide (11326040):** Yield 48%, M.p. 120-2 °C, ^{1}H NMR (300 MHz, DMSO-d6): 8.56 (br s, 1H), 7.64 (s, 1H), 7.39 (d, 1H, *J* = 3.7 Hz), 7.12 (d, 2H, *J* = 8.7 Hz), 6.97 (d, 2H, *J* = 8.7 Hz), 6.79 (d, 1H, *J* = 3.7 Hz), 4.08 (q, 2H, *J* = 6.9 Hz), 3.67 (t, 2H, *J* = 7.1 Hz), 2.78 (t, 2H, *J* = 7.1 Hz), 2.40 (s, 3H), 1.38 (t, 3H, *J* = 6.9 Hz); LCMS (ESI): *m/z* 317.4 $(M+H)^+$.

***N*-[(1*E*)-(1-Adamantylamino)methylene]-5-methylthiophene-2-carboxamide (11326041):** Yield 51%, M.p. 170-2 °C, ^{1}H NMR (300 MHz, DMSO-d6): 7.71 (s, 1H), 7.45 (d, 1H, *J* = 3.7 Hz), 7.06 (br s, 1H), 6.74 (d, 1H, *J* = 3.7 Hz), 2.42 (s, 3H), 2.18 (br s, 3H), 1.91 (br s, 3H), 1.69 (br s, 3H); LCMS (ESI): *m/z* 303.4 $(M+H)^+$.

***N*-(2,3-Difluorophenyl)-5-methyl-4-nitrothiophene-2-carboxamide (11326042):** Yield 68%, M.p. 150-2 °C, ^{1}H NMR (300 MHz, DMSO-d6): 9.73 (br s, 1H), 8.12 (s, 1H), 7.57 (m, 1H), 6.58 (m, 2H), 2.73 (s, 3H); LCMS (ESI): *m/z* 299.3 $(M+H)^+$.

5-Methyl-4-nitro-*N*-(4-nitrophenyl)thiophene-2-carboxamide (11326043): Yield 61%, M.p. 273-5 °C, ^{1}H NMR (300 MHz, DMSO-d6): 9.12 (br s, 1H), 8.23 (d, 2H, *J* = 10.4 Hz), 8.11 (s, 1H), 7.75 (d, 2H, *J* = 10.4 Hz), 2.73 (s, 3H); LCMS (ESI): *m/z* 308.3 $(M+H)^+$.

5-Methyl-4-nitro-*N*-[4-(trifluoromethyl)phenyl]thiophene-2-carboxamide (11326044): Yield 64%, M.p. 260-2 °C, ^{1}H NMR (300 MHz, DMSO-d6): 9.49 (br s, 1H), 8.12 (s, 1H), 7.76 (d, 2H, *J* = 8.4 Hz), 2.69 (s, 3H); LCMS (ESI): *m/z* 331.3 $(M+H)^+$.

***N*,5-Dimethyl-*N*-(4-methylphenyl)-4-nitrothiophene-2-carboxamide (11326045):** Yield 73%, M.p. 140-2 °C, ^{1}H NMR (300 MHz, DMSO-d6): 8.05 (s, 1H), 7.19 (m, 4H), 3.68 (s, 3H), 2.74 (s, 3H), 2.35 (s, 3H); LCMS (ESI): *m/z* 291.3 $(M+H)^+$.

***N*-[2-(4-Methoxyphenyl)ethyl]-5-methyl-4-nitrothiophene-2-carboxamide (11326046):** Yield 52%, M.p. 148-50 °C, ^{1}H NMR (300 MHz, DMSO-d6): 8.12 (s, 1H), 7.16 (d, 2H, *J* = 8.6 Hz), 6.94 (br s, 1H), 6.87 (d, 2H, *J* = 8.6 Hz), 4.02 (q, 2H, *J* = 6.9 Hz), 3.43 (t, 2H, *J* = 7.1 Hz), 2.74 (t, 2H, *J* = 7.1 Hz), 2.63 (s, 3H), 1.41 (t, 3H, *J* = 6.9 Hz); LCMS (ESI): *m/z* 321.4 $(M+H)^+$.

***N*-1-Adamantyl-5-methyl-4-nitrothiophene-2-carboxamide (11326047):** Yield 49%, M.p. 190-2 °C, ^{1}H NMR (300 MHz, DMSO-d6): 8.23 (s, 1H), 4.66 (br s, 1H), 2.69 (s, 3H), 2.01 (br s, 3H), 1.67 (m, 12H); LCMS (ESI): *m/z* 321.4 $(M+H)^+$.

***N*-2-Adamantyl-5-methyl-4-nitrothiophene-2-carboxamide (11326048):** Yield 52%, M.p. 178-80 °C, ^{1}H NMR (300 MHz, DMSO-d6): 8.21 (s, 1H), 6.70 (br s, 1H), 3.98 (m, 1H), 2.74 (s, 3H), 1.93-1.42 (m, 14H); LCMS (ESI): *m/z* 321.4 $(M+H)^+$.

***N*-[(1*E*)-(2-Adamantylamino)methylene]-5-methyl-4-nitrothiophene-2-carboxamide (11326049):** Yield 16%, M.p. 134-7 °C, ^{1}H NMR (300 MHz, DMSO-d6): 8.15 (br s, 1H), 8.05 (s, 1H), 7.51 (s, 1H), 2.94 (m, 1H), 2.70 (s, 3H), 1.78-1.17 (m, 14H); LCMS (ESI): *m/z* 348.5 $(M+H)^+$.

***N*-(4-Fluorophenyl)-5-methyl-4-nitrothiophene-2-carboxamide (11326050):** Yield 59%, M.p. 208-10 ^oC, ^1H NMR (300 MHz, DMSO-d6): 9.23 (br s, 1H), 8.12 (s, 1H), 7.64 (m, 2H), 6.97 (m, 2H), 2.74 (s, 3H); LCMS (ESI): *m/z* 281.3 $(M+H)^+$.

***N*-{(1*E*)-[(4-Fluorophenyl)amino]methylene}-5-methylthiophene-2-carboxamide (11326051):** Yield 53%, M.p. 162-4 ^oC, ^1H NMR (300 MHz, DMSO-d6): 10.51 (br s, 1H), 7.64 (d, 1H, *J* = 3.6 Hz), 7.32 (m, 2H), 7.16 (s, 1H), 6.93 (m, 2H), 6.83 (d, 1H, *J* = 3.6 Hz), 2.42 (s, 3H); LCMS (ESI): *m/z* 263,3 $(M+H)^+$.

***N*-{(1*E*)-[(3,4-dimethoxyphenyl)amino]methylene}-5-methylthiophene-2-carboxamide (11326052):** Yield 49%, M.p. 162-4 ^oC, ^1H NMR (300 MHz, DMSO-d6): 11.28 (br s, 1H), 7.60 (d, 1H, *J* = 3.5 Hz), 7.11 (s, 1H), 7.02 (m, 2H), 6.89 (d, 1H, *J* = 3.5 Hz), 6.68 (s, 1H), 4.03 (s, 3H), 3.87 (s, 3H), 2.44 (s, 3H); LCMS (ESI): *m/z* 305.4 $(M+H)^+$.

***N*-[(1*Z*)-3,4-dihydroisoquinolin-2(1*H*)-ylmethylene]-5-methylthiophene-2-carboxamide (11326053):** Yield 54%, M.p. 125-7 ^oC, ^1H NMR (300 MHz, DMSO-d6): 7.83 (s, 1H), 7.54 (d, 1H, *J* = 3.5 Hz), 7.28 (m, 2H), 6.98 (m, 2H), 6.86 (d, 1H, *J* = 3.5 Hz), 4.34 (q, 2H, *J* = 15.0 Hz), 3.53 (m, 2H), 3.16 (m, 2H), 2.48 (s, 3H); LCMS (ESI): *m/z* 285.4 $(M+H)^+$.

***N*-(3,4-Dimethoxyphenyl)-5-methyl-4-nitrothiophene-2-carboxamide (11326054):** Yield 65%, M.p. 200-2 ^oC, ^1H NMR (300 MHz, DMSO-d6): 10.32 (br s, 1H), 8.12 (s, 1H), 7.47 (s, 1H), 7.12 (d, 1H, *J* = 8.5 Hz), 6.83 (d, 1H, *J* = 8.5 Hz), 4.04 (s, 3H), 3.90 (s, 3H), 2.72 (s, 3H); LCMS (ESI): *m/z* 322.3 $(M+H)^+$.

1-[(5-Methyl-4-nitro-2-thienyl)carbonyl]-1,2,3,4-tetrahydroquinoline (11326055): Yield 61%, M.p. 85-7 ^oC, ^1H NMR (300 MHz, DMSO-d6): 7.96 (s, 1H), 7.61 (m, 1H), 7.14 (m, 3H), 4.73 (q, 2H, *J* = 15.0 Hz), 3.87 (m, 2H), 3.05 (m, 2H), 2.69 (s, 3H); LCMS (ESI): *m/z* 303.4 $(M+H)^+$.

5-Methyl-*N*-(4-methylphenyl)thiophene-2-carboxamide (11326097): Yield 51%, M.p. 156-8^{0}C, ^1H NMR (300 MHz, DMSO-d6): 9.23 (br s, 1H), 7.48 (d, 1H, *J* = 3.5 Hz), 7.40 (d, 2H, *J* = 8.0 Hz), 7.10 (d, 2H, *J* = 8.0 Hz), 6.81 (d, 1H, *J* = 3.5 Hz), 2.46 (s, 3H), 2.28 (s, 3H); LCMS (ESI): *m/z* 232.3 $(M+H)^+$.

***N*-(4-Fluorophenyl)-2-furamide (11326098):** Yield 61%, M.p. 102-4 ^oC, ^1H NMR (300 MHz, DMSO-d6): δ 9.01 (br s, 1H), 7.63 (m, 2H), 7.41 (q, 1H, *J* = 0.8 Hz), 7.16 (d, 1H, *J* = 3.3 Hz), 6.97 (m, 2H), 6.43 (d, 1H, *J* = 3.3 Hz); LCMS (ESI): *m/z* 206.2 $(M+H)^+$.

***N*-(3-Fluorophenyl)-5-methylthiophene-2-carboxamide (11326099):** Yield 57%, M.p. 173-6 ^oC, ^1H NMR (300 MHz, DMSO-d6): δ 9.38 (br s, 1H), 7.68 (m, 1H), 7.43 (d, 1H, *J* = 3.5 Hz), 7.37 (m, 1H), 6.83 (m, 2H), 6.78 (d, 1H, *J* = 3.5 Hz), 2.43 (s, 3H); LCMS (ESI): *m/z* 236.3 $(M+H)^+$.

***N*-(3,5-Difluorophenyl)-5-methylthiophene-2-carboxamide (11326100):** Yield 65%, M.p. 151-3 ^{o}C, ^{1}H NMR (300 MHz, DMSO-d6): δ 9.82 (br s, 1H), 7.54 (m, 2H), 7.44 (d, 1H, *J* = 3.5 Hz), 6.81 (d, 1H, *J* = 3.5 Hz), 6.56 (m, 1H), 2.44 (s, 3H); LCMS (ESI): *m/z* 254.3 $(M+H)^+$.

***N*-(3,4-Difluorophenyl)-5-methylthiophene-2-carboxamide (11326101):** Yield 68%, M.p. 128-30 ^{o}C, ^{1}H NMR (300 MHz, DMSO-d6): δ 9.31 (br s, 1H), 7.61 (m, 2H), 7.48 (d, 1H, *J* = 3.5 Hz), 7.42 (m, 2H), 7.18 (m, 2H), 6.79 (d, 1H, *J* = 3.5 Hz), 2.39 (s, 3H); LCMS (ESI): *m/z* 254.3 $(M+H)^+$.

***N*-(3-Fluorophenyl)-4,5-dimethylthiophene-2-carboxamide (11326102):** Yield 60%, M.p. 138-40 ^{o}C, ^{1}H NMR (300 MHz, DMSO-d6): δ 9.17 (br s, 1H), 7.64 (m, 2H), 6.97 (m, 2H), 6.89 (s, 1H), 2.35 (s, 3H), 2.16 (s, 3H); LCMS (ESI): *m/z* 250.3 $(M+H)^+$.

***N*-(4-Chlorophenyl)-5-methylthiophene-2-carboxamide (11326103):** Yield 71%, M.p. 142-4 ^{o}C, ^{1}H NMR (300 MHz, DMSO-d6): δ 9.21 (br s, 1H), 7.56 (d, 2H, *J* = 9.3 Hz), 7.46 (d, 1H, *J* = 3.5 Hz), 7.31 (d, 2H, *J* = 9.3 Hz), 6.78 (d, 1H, *J* = 3.5 Hz), 2.38 (s, 3H); LCMS (ESI): *m/z* 252.7 $(M+H)^+$.

***N*-(4-Chlorobenzyl)-5-methylthiophene-2-carboxamide (11326104):** Yield 74%, M.p. 148-50 ^{o}C, ^{1}H NMR (300 MHz, DMSO-d6): δ 7.76 (br s, 1H), 7.41 (d, 1H, *J* = 3.5 Hz), 7.25 (d, 2H, *J* = 9.1 Hz), 7.13 (d, 2H, *J* = 9.1 Hz), 6.92 (d, 1H, *J* = 3.5 Hz), 4.43 (d, 2H, *J* = 0.4 Hz), 2.42 (s, 3H); LCMS (ESI): *m/z* 266.8 $(M+H)^+$.

***N*-(4-Chloropyridin-2-yl)-5-methylthiophene-2-carboxamide (11326105):):** Yield 69%, M.p. 128-30 ^{o}C, ^{1}H NMR (300 MHz, DMSO-d6): δ 9.56 (br s, 1H), 8.37 (d, 1H, *J* = 6.2 Hz), 7.78 (s, 1H), 7.47 (d, 1H, *J* = 3.5 Hz), 7.13 (d, 1H, *J* = 6.2 Hz), 6.79 (d, 1H, *J* = 3.5 Hz), 2.44 (s, 3H); LCMS (ESI): *m/z* 253.7 $(M+H)^+$.

***N*-(5-Chloropyridin-2-yl)-5-methylthiophene-2-carboxamide (11326106):** Yield 71%, M.p. 140-2 ^{o}C, ^{1}H NMR (300 MHz, DMSO-d6): δ 9.04 (br s, 1H), 8.29 (d, 1H, *J* = 8.9 Hz), 8.16 (s, 1H), 7.93 (d, 1H, *J* = 8.9 Hz), 7.47 (d, 1H, *J* = 3.5 Hz), 6.76 (d, 1H, *J* = 3.5 Hz), 2.43 (s, 3H); LCMS (ESI): *m/z* 253.7 $(M+H)^+$.

***N*-(3,4-Dimethoxyphenyl)-2-furamide (10326107):** Yield 57%, M.p. 135-7 ^{o}C, ^{1}H NMR (300 MHz, DMSO-d6): δ 9.47 (br s, 1H), 7.47 (s, 1H), 7.39 (q, 1H, *J* = 0.8 Hz), 7.16 (d, 1H, *J* = 3.3 Hz), 7.13 (d, 1H, *J* = 8.3 Hz), 6.84 (d, 2H, *J* = 8.3 Hz), 6.42 (d, 1H, *J* = 2.2 Hz), 3.98 (s, 3H), 3.84 (s, 3H); LCMS (ESI): *m/z* 248.3 $(M+H)^+$.

***N*-(3,4-Dimethoxyphenyl)-4,5-dimethylthiophene-2-carboxamide (11326108):** Yield 69%, M.p. 158-60 ^{o}C, ^{1}H NMR (300 MHz, DMSO-d6): δ 10.02 (br s, 1H), 7.48 (s, 1H), 7.12 (d, 2H, *J* = 8.5 Hz), 6.90 (s, 1H), 6.83 (d, 2H, *J* = 8.5 Hz), 4.08 (s, 3H), 3.97 (s, 3H), 2.35 (s, 3H), 2.17 (s, 3H); LCMS (ESI): *m/z* 292.4 $(M+H)^+$.

5-Methyl-*N*-{2-[4-(methylthio)phenyl]ethyl}thiophene-2-carboxamide (11326113): Yield 74%, M.p. 118-20 ^{o}C, ^{1}H NMR (300 MHz, DMSO-d6): δ 7.43 (d, 1H, *J* = 3.5 Hz), 7.24 (d, 2H, *J* = 8.5 Hz), 7.03 (d, 2H, *J* = 8.5 Hz), 6.94 (br s, 1H), 6.89 (d, 1H, *J* = 3.5 Hz), 3.46 (t, 2H, *J* = 6.3 Hz), 2.85 (t, 2H, *J* = 6.3 Hz), 2.43 (s, 3H); LCMS (ESI): *m/z* 292.2 $(M+H)^{+}$.

***N*-(4-Fluorophenyl)thiophene-2-carboxamide (11326193):** Yield 71%, M.p. 118-20 ^{o}C, ^{1}H NMR (300 MHz, DMSO-d6): 9.23 (br s, 1H), 7.83 (d, 1H, *J* = 3.5 Hz), 7.68 (d, 1H, *J* = 3.5 Hz), 7.63 (m, 2H), 7.15 (dd, 1H, *J* = 4.5 Hz, *J* = 0.9 Hz), 6.98 (m, 2H); LCMS (ESI): *m/z* 222.3 $(M+H)^{+}$.

***N*-(3,4-Difluorophenyl)thiophene-2-carboxamide (11326194):** Yield 69%, M.p. 119-21 ^{o}C, ^{1}H NMR (300 MHz, DMSO-d6): 9.51 (br s, 1H), 7.89 (d, 1H, *J* = 3.5 Hz), 7.70 (d, 1H, *J* = 3.5 Hz), 7.60 (br s, 1H), 7.45 (br s, 1H), 7.21 (br s, 1H), 7.15 (dd, 1H, *J* = 4.5 Hz, *J* = 0.9 Hz); LCMS (ESI): *m/z* 240.2 $(M+H)^{+}$.

***N*-(3,4-Difluorophenyl)-3-methylthiophene-2-carboxamide (11326195):** Yield 65%, M.p. 116-8 ^{o}C, ^{1}H NMR (300 MHz, DMSO-d6): 9.43 (br s, 1H), 7.58 (br s, 1H), 7.43 (br s, 1H), 7.25 (d, 1H, *J* = 5.1 Hz), 7.18 (br s, 1H), 6.94 (d, 1H, *J* = 5.1 Hz), 2.27 (s, 3H); LCMS (ESI): *m/z* 254.3 $(M+H)^{+}$.

***N*-(3,5-Difluorophenyl)thiophene-2-carboxamide (11326196):** Yield 68%, M.p. 103-5 ^{o}C, ^{1}H NMR (300 MHz, DMSO-d6): 9.82 (br s, 1H), 7.84 (d, 1H, *J* = 3.5 Hz), 7.72 (d, 1H, *J* = 3.5 Hz), 7.52 (s, 2H), 7.14 (dd, 1H, *J* = 4.5 Hz, *J* = 0.9 Hz), 6,54 (s, 1H); LCMS (ESI): *m/z* 240.2 $(M+H)^{+}$.

***N*-(3,5-Difluorophenyl)-3-methylthiophene-2-carboxamide (11326197):** Yield 70%, M.p. 127-9 ^{o}C, ^{1}H NMR (300 MHz, DMSO-d6): 9.73 (br s, 1H), 7.53 (m, 2H), 7.26 (d, 1H, *J* = 5.1 Hz), 6.94 (d, 1H, *J* = 5.1 Hz), 6.57 (m, 1H), 7.14 (dd, 1H, *J* = 4.5 Hz, *J* = 0.9 Hz), 6,54 (s, 1H); LCMS (ESI): *m/z* 254.3 $(M+H)^{+}$.

***N*-(4-Chlorophenyl)thiophene-2-carboxamide (11326198):** Yield 71%, M.p. 154-6 ^{o}C, ^{1}H NMR (300 MHz, DMSO-d6): 9.82 (br s, 1H), 7.80 (d, 1H, *J* = 3.5 Hz), 7.69 (d, 1H, *J* = 3.5 Hz), 7.54 (d, 2H, *J* = 9.4 Hz), 7.35 (d, 2H, *J* = 9.4 Hz), 2.27 (s, 3H); LCMS (ESI): *m/z* 238.7 $(M+H)^{+}$.

***N*-(4-Chlorophenyl)-3-methylthiophene-2-carboxamide (11326199):** Yield 64%, M.p. 125-7 ^{o}C, ^{1}H NMR (300 MHz, DMSO-d6): 9.11 (br s, 1H), 7.54 (d, 2H, *J* = 9.5 Hz), 7.35 (d, 2H, *J* = 9.5 Hz), 7.26 (d, 1H, *J* = 5.1 Hz), 6.92 (d, 1H, *J* = 5.1 Hz), 2.26 (s, 3H); LCMS (ESI): *m/z* 252.7 $(M+H)^{+}$.

***N*-(4-Fluorophenyl)-5-methyl-2-furamide (11326200):** Yield 74%, M.p. 86-8 ^{o}C, ^{1}H NMR (300 MHz, DMSO-d6): δ 9.24 (br s, 1H), 7.64 (m, 2H), 7.02 (d, 1H, *J* = 3.4 Hz), 6.95 (m, 2H), 6.15 (d, 1H, *J* = 3.4 Hz), 2.28 (s, 3H); LCMS (ESI): *m/z* 220.2 $(M+H)^{+}$.

***N*-(3,4-Difluorophenyl)-5-methyl-2-furamide (11326201):** Yield 56%, M.p. 88-90 ^{o}C, ^{1}H NMR (300 MHz, DMSO-d6): δ 9.32 (br s, 1H), 7.63 (br s, 1H), 7.45 (br s, 1H), 7.18 (br s, 1H), 7.01 (d, 1H, *J* = 3.4 Hz), 6.14 (d, 1H, *J* = 3.4 Hz), 2.27 (s, 3H); LCMS (ESI): *m/z* 238.2 $(M+H)^{+}$.

***N*-(3,5-Difluorophenyl)-5-methyl-2-furamide (11326202):** Yield 63%, M.p. 62-4 ^{o} C, ^{1}H NMR (300 MHz, DMSO-d6): δ 9.61 (br s, 1H), 7.52 (br s, 2H), 7.01 (d, 1H, *J* = 3.4 Hz), 7.55 (br s, 2H), 6.14 (d, 1H, *J* = 3.4 Hz), 2.28 (s, 3H); LCMS (ESI): *m/z* 238.2 $(M+H)^+$.

***N*-(3,4-Difluorophenyl)-4,5-dimethylthiophene-2-carboxamide (11326203):** Yield 62%, M.p. 153-5 ^{o}C, ^{1}H NMR (300 MHz, DMSO-d6): δ 9.46 (br s, 1H), 7.57 (br s, 1H), 7.42 (m, 1H), 7.23 (s, 1H), 6.99 (s, 1H), 2.35 (s, 3H), 2.16 (s, 3H); LCMS (ESI): *m/z* 268,3 $(M+H)^+$.

***N*-(4-Chlorophenyl)-5-methyl-2-furamide (11326204):** Yield 79%, M.p. 128-30 ^{o} C, ^{1}H NMR (300 MHz, DMSO-d6): δ 9.15 (br s, 1H), 7.55 (br s, 2H), 7.32 (br s, 2H), 7.00 (d, 1H, *J* = 3.4 Hz), 6.14 (d, 1H, *J* = 3.4 Hz), 2.29 (s, 3H); LCMS (ESI): *m/z* 236.7 $(M+H)^+$.

***N*-(3,4-Dimethoxyphenyl)-5-methyl-2-furamide (11326205):** Yield 74%, M.p. 116-8 ^{o} C, ^{1}H NMR (300 MHz, DMSO-d6): δ 9.87 (br s, 1H), 7.47 (s, 1H), 7.12 (d, 1H, *J* = 9.4 Hz), 7.02 (d, 1H, *J* = 3.4 Hz), 6.85 (d, 1H, *J* = 9.4 Hz), 6.13 (d, 1H, *J* = 3.4 Hz), 3.98 (s, 3H), 3.83 (s, 3H), 2.29 (s, 3H); LCMS (ESI): *m/z* 262.3 $(M+H)^+$.

***N*-(3,4-Dimethoxyphenyl)thiophene-2-carboxamide (11326206):** Yield 74%, M.p. 175-7 ^{o}C, ^{1}H NMR (300 MHz, DMSO-d6): 10.12 (br s, 1H), 7.89 (d, 1H, *J* = 3.5 Hz), 7.12 (d, 1H, *J* = 9.7 Hz), 6.84 (d, 1H, *J* = 9.7 Hz), 3.99 (s, 3H), 3.83 (s, 3H); LCMS (ESI): *m/z* 264.3 $(M+H)^+$.

***N*-(3,4-Dimethoxyphenyl)-3-methylthiophene-2-carboxamide (11326207):** Yield 62%, M.p. 138-40 ^{o}C, ^{1}H NMR (300 MHz, DMSO-d6): 10.17 (br s, 1H), 7.44 (s, 1H), 7.25 (d, 1H, *J* = 4.7 Hz), 7.11 (d, 1H, *J* = 9.7 Hz), 6.92 (d, 1H, *J* = 4.7 Hz), 6.85 (d, 1H, *J* = 9.7 Hz), 4.08 (s, 3H), 3.92 (s, 3H), 2.25 (s, 1H); LCMS (ESI): *m/z* 278.3 $(M+H)^+$.

***N*-(3,5-Difluorophenyl)-4,5-dimethylthiophene-2-carboxamide (11326208):** Yield 58%, M.p. 138-40 ^{o}C, ^{1}H NMR (300 MHz, DMSO-d6): 9.72 (br s, 1H), 7.55 (m, 2H), 6.93 (s, 1H), 6.56 (m, 1H), 2.35 (s, 3H), 2.16 (s, 3H); LCMS (ESI): *m/z* 268.3 $(M+H)^+$.

***N*-(4-Chlorophenyl)-4,5-dimethylthiophene-2-carboxamide (11326210):** Yield 63%, M.p. 148-50 ^{o}C, ^{1}H NMR (300 MHz, DMSO-d6): δ 9.27 (br s, 1H), 7.53 (d, 2H, *J* = 9.5 Hz), 7.35 (d, 2H, *J* = 9.5 Hz), 6.98 (s, 1H), 2.35 (s, 3H), 2.16 (s, 3H); LCMS (ESI): *m/z* 266.8 $(M+H)^+$.

***N*-(4-Fluorophenyl)-4-methylthiophene-2-carboxamide (11326211):** Yield 68%, M.p. 125-7 ^{o}C, ^{1}H NMR (300 MHz, DMSO-d6): δ 9.31 (br s, 1H), 7.67 (m, 2H), 7.06 (s, 1H), 6.97 (m, 2H), 6.91 (s, 1H), 2.15 (s, 3H); LCMS (ESI): *m/z* 236.3 $(M+H)^+$.

***N*-(3,4-Difluorophenyl)-4-methylthiophene-2-carboxamide (11326212):** Yield 60%, M.p. 150-2 ^{o}C, ^{1}H NMR (300 MHz, DMSO-d6): δ 9.46 (br s, 1H), 7.60 (m, 1H), 7.41 (m, 1H), 7.17 (m, 1H), 7.07 (s, 1H), 6.92 (s, 1H), 2.14 (s, 3H); LCMS (ESI): *m/z* 254.3 $(M+H)^+$.

***N*-(3,5-Difluorophenyl)-4-methylthiophene-2-carboxamide (11326213):** Yield 60%, M.p. 125-7 ^{o}C, ^{1}H NMR (300 MHz, DMSO-d6): δ 9.81 (br s, 1H), 7.51 (m, 2H), 7.06 (s, 1H), 6.92 (s, 1H), 6.56 (m, 1H), 2.16 (s, 3H); LCMS (ESI): *m/z* 254.3 $(M+H)^{+}$.

***N*-(3,4-Dimethoxyphenyl)-4-methylthiophene-2-carboxamide (11326214):** Yield 68%, M.p. 126-8 ^{o}C, ^{1}H NMR (300 MHz, DMSO-d6): δ 10.25 (br s, 1H), 7.47 (s, 1H), 7.12 (s, 1H, *J* = 8.6 Hz), 7.06 (s, 1H), 6.93 (s, 1H), 6.81 (s, 1H, *J* = 8.6 Hz), 4.03 (s, 3H), 3.88 (s, 3H), 2.17 (s, 1H); LCMS (ESI): *m/z* 278.3 $(M+H)^{+}$.

***N*-(4-Chlorophenyl)-4-methylthiophene-2-carboxamide (11326215):** Yield 65%, M.p. 185-7 ^{o}C, ^{1}H NMR (300 MHz, DMSO-d6): δ 9.22 (br s, 1H), 7.54 (d, 2H, *J* = 9.5 Hz), 7.35 (d, 2H, *J* = 9.5 Hz), 7.06 (s, 1H), 6.93 (s, 1H), 2.16 (s, 3H); LCMS (ESI): *m/z* 252.7 $(M+H)^{+}$.

***N*-(4-Fluorophenyl)-4-methyl-1,3-thiazole-5-carboxamide (11326217):** Yield 49%, M.p. 129-31 ^{o}C, ^{1}H NMR (300 MHz, DMSO-d6): δ 9.89 (br s, 1H), 8.77 (s, 1H), 7.71 (m, 2H), 6.96 (m, 2H), 2.41 (s, 3H); LCMS (ESI): *m/z* 237.3 $(M+H)^{+}$.

***N*-(3,5-Difluorophenyl)-4-methyl-1,3-thiazole-5-carboxamide (11326218):** Yield 74%, M.p. 122-24 ^{o}C, ^{1}H NMR (300 MHz, DMSO-d6): δ 10.54 (br s, 1H), 8.76 (s, 1H), 7.59 (m, 2H), 6.56 (m, 1H), 2.40 (s, 3H); LCMS (ESI): *m/z* 255.3 $(M+H)^{+}$.

***N*-(4-Chlorophenyl)-4-methyl-1,3-thiazole-5-carboxamide (11326220):** Yield 74%, M.p. 135-37 ^{o}C, ^{1}H NMR (300 MHz, DMSO-d6): δ 9.93 (br s, 1H), 8.77 (s, 1H), 7.61 (m, 2H), 7.32 (m, 2H), 2.43 (s, 3H); LCMS (ESI): *m/z* 253,7 $(M+H)^{+}$.

1-Ethyl-*N*-(4-fluorophenyl)-3-methyl-1*H*-pyrazole-5-carboxamide (11326221): Yield 70%, M.p. 65-7 ^{o}C, ^{1}H NMR (300 MHz, DMSO-d6): δ 10.43 (br s, 1H), 7.81 (m, 2H), 6.99 (m, 2H), 6.62 (s, 1H), 4.42 (q, 2H, *J* = 7.4 Hz), 2.23 (s, 3H), 1.39 (t, 3H, d, *J* = 7.4 Hz); LCMS (ESI): *m/z* 248.3 $(M+H)^{+}$.

***N*-(3,4-Difluorophenyl)-1-ethyl-3-methyl-1*H*-pyrazole-5-carboxamide (11326222):** Yield 70%, M.p. 126-28 ^{o}C, ^{1}H NMR (300 MHz, DMSO-d6): δ 10.43 (br s, 1H), 7.76 (br s, 1H), 7.60 (br s, 1H), 7.22 (br s, 1H), 6.61 (s, 1H), 4.44 (q, 2H, *J* = 7.4 Hz), 2.25 (s, 3H), 1.40 (t, 3H, d, *J* = 7.4 Hz); LCMS (ESI): *m/z* 266.3 $(M+H)^{+}$.

***N*-(3,5-Difluorophenyl)-1-ethyl-3-methyl-1*H*-pyrazole-5-carboxamide (11326223):** Yield 65%, M.p. 124-26 ^{o}C, ^{1}H NMR (300 MHz, DMSO-d6): δ 10.53 (br s, 1H), 7.68 (m, 2H), 6.61 (s, 1H), 6.56 (br s, 1H), 4.42 (q, 2H, *J* = 7.4 Hz), 2.25 (s, 3H), 1.39 (t, 3H, d, *J* = 7.4 Hz); LCMS (ESI): *m/z* 266.3 $(M+H)^{+}$.

***N*-(3,4-Dimethoxyphenyl)-1-ethyl-3-methyl-1*H*-pyrazole-5-carboxamide (11326224):** Yield 62%, M.p. 118-20 ^{o}C, ^{1}H NMR (300 MHz, DMSO-d6): δ 9.87 (br s, 1H), 7.63 (s, 1H), 7.26 (d, 1H,

J = 9.4 Hz), 6.89 (d, 1H, *J* = 9.4 Hz), 6.61 (s, 1H), 4.48 (q, 2H, *J* = 7.4 Hz), 3.98 (s, 3H), 3.83 (s, 3H), 2.25 (s, 3H), 1.39 (t, 3H, d, *J* = 7.4 Hz); LCMS (ESI): *m/z* 290.3 $(M+H)^+$.

***N*-(4-Chlorophenyl)-1-ethyl-3-methyl-1*H*-pyrazole-5-carboxamide (11326225):** Yield 59%, M.p. 92-4 ^{o}C, ^{1}H NMR (300 MHz, DMSO-d6): δ 9.95 (br s, 1H), 7.54 (m, 2H), 7.37 (m, 2H), 6.60 (s, 1H), 4.41 (q, 2H, *J* = 7.4 Hz), 2.23 (s, 3H), 1.40 (t, 3H, d, *J* = 7.4 Hz); LCMS (ESI): *m/z* 264.7 $(M+H)^+$.

***N*-(4-Fluorophenyl)-3-methylthiophene-2-carboxamide (11326232):** Yield 64%, M.p. 121-3 ^{o}C, ^{1}H NMR (300 MHz, DMSO-d6): δ 9.09 (br s, 1H), 7.63 (s, 1H), 7.25 (d, 1H, *J* = 4.7 Hz), 6.99 (m, 2H), 6.92 (d, 1H, *J* = 4.7 Hz), 2.24 (s, 3H); LCMS (ESI): *m/z* 236.3 $(M+H)^+$.

5-Methyl-*N*-(4-nitrophenyl)thiophene-2-carboxamide 1,1-dioxide (11426026): Yield 64%, M.p. 178-80 ^{o}C, ^{1}H NMR (300 MHz, DMSO-d6): δ 8.81 (d, 1H, *J* = 3.1 Hz), 8.61 (br s, 1H), 8.55 (d, 2H, *J* = 10.4 Hz), 8.07 (d, 2H, *J* = 10.4 Hz), 7.15 (d, 1H, *J* = 3.1 Hz), 2.32 (s, 3H); LCMS (ESI): *m/z* 295.3 $(M+H)^+$.

***N*-(4-Fluorophenyl)-4,5-dimethylthiophene-2-carboxamide 1,1-dioxide (11426169):** Yield 56%, M.p. 190-92 ^{o}C, ^{1}H NMR (300 MHz, DMSO-d6): δ 9.01 (s, 1H), 8.74 (br s, 1H), 7.93 (m, 2H), 7.29 (m, 2H), 2.88 (s, 3H), 2.19 (s, 3H); LCMS (ESI): *m/z* 282.3 $(M+H)^+$.

5-Methyl-*N*-(3,4,5-trifluorophenyl)thiophene-2-carboxamide 1,1-dioxide (11426170): Yield 58 %, M.p. 230-34 ^{o}C, ^{1}H NMR (300 MHz, DMSO-d6): δ 9.26 (br s, 1H), 8.76 (d, 1H, *J* = 3.1 Hz), 7.76 (m, 2H), 7.13 (d, 1H, *J* = 3.1 Hz), 2.42 (s, 3H); LCMS (ESI): *m/z* 304.3 $(M+H)^+$.

5-Methyl-*N*-[4-(trifluoromethyl)phenyl]thiophene-2-carboxamide 1,1-dioxide (11426171): Yield 63 %, M.p. 238-40 ^{o}C, ^{1}H NMR (300 MHz, DMSO-d6): δ 8.80 (d, 1H, *J* = 3.1 Hz), 8.63 (br s, 1H), 8.07 (d, 2H, *J* = 8.5 Hz), 7.99 (d, 2H, *J* = 8.5 Hz), 7.14 (d, 1H, *J* = 3.1 Hz), 2.29 (s, 3H); LCMS (ESI): *m/z* 318,3 $(M+H)^+$.

***N*-(4-Chlorophenyl)-5-methylthiophene-2-carboxamide 1,1-dioxide (11426172):** Yield 68 %, M.p. 235-37 ^{o}C, ^{1}H NMR (300 MHz, DMSO-d6): δ 8.83 (d, 1H, *J* = 3.1 Hz), 8.70 (br s, 1H), 7.83 (d, 2H, *J* = 9.6 Hz), 7.64 (d, 2H, *J* = 9.6 Hz), 7.18 (d, 1H, *J* = 3.1 Hz), 2.29 (s, 3H); LCMS (ESI): *m/z* 284.7 $(M+H)^+$.

***N*-(3,4-Difluorophenyl)-5-methylthiophene-2-carboxamide 1,1-dioxide (11426173):** Yield 64 %, M.p. 225-27 ^{o}C, ^{1}H NMR (300 MHz, DMSO-d6): δ 8.96 (br s, 1H), 8.80 (d, 1H, *J* = 3.1 Hz), 7.86 (m, 1H), 7.74 (m, 1H), 7.49 (m, 1H), 7.14 (d, 1H, *J* = 3.1 Hz), 2.34 (s, 3H); LCMS (ESI): *m/z* 286.3 $(M+H)^+$.

5-Methylthiophen-2(3*H*)-one 1,1-dioxide (11426174): Yield 73%, M.p. 134-36 ^{o}C, ^{1}H NMR (300 MHz, DMSO-d6): δ 6.09 (d, 1H, *J* = 1.5 Hz), 3.42 (t, 1H, *J* = 2.5 Hz), 1.96 (s, 1H); LCMS

LCMS (ESI): *m/z* 147.2 $(M+H)^+$.

SUPPLEMENTARY REFERENCES

Copeland, A.R. (2000). Enzymes: A Practical Introduction to Structure, Mechanism, and Data Analysis.

King, J.Y., Ferrara, R., Tabibiazar, R., Spin, J.M., Chen, M.M., Kuchinsky, A., Vailaya, A., Kincaid, R., Tsalenko, A., Deng, D.X., et al. (2005). Pathway analysis of coronary atherosclerosis. Physiol. Genomics. *23*, 103-118.

Manganelli, R., Voskuil, M.I., Schoolnik, G.K., and Smith, I. (2001). The *Mycobacterium tuberculosis* ECF sigma factor sigmaE: role in global gene expression and survival in macrophages. Mol. Microbiol. *41*, 423-37.

Pesek, J.J., Matyska, M.T., Fischer, S.M., and Sana, T.R. (2008). Analysis of hydrophilic metabolites by high-performance liquid chromatography-mass spectrometry using a silica hydride-based stationary phase. J. Chromatogr. A. *1204*, 48-55.

Rao, S.N., Head, M.S., Kulkarni, A., LaLonde, J.M. (2007). Validation studies of the site-directed docking program LibDock. J. Chem. Inf. Model. *47*, 2159-2171.

Robert, X., and Gouet, P. (2014) Deciphering key features in protein structures with the new ENDscript server. Nucleic Acids Res. *42*, W320-4.

Sambrook, J., and Russel, D.W. (2001). Molecular Cloning: a laboratory manual.

Singh, V., Brecik, M., Mukherjee, R., Evans, J.C., Svetlikova, Z., Blasko, J., Surade, S., Blackburn, J., Warner, D.F., Mikusova, K., and Mizrahi, V. (2015). The complex mechanism of antimycobacterial action of 5-Fluorouracil. Chem. Biol. *22*, 63-75.

Stierand, K., and Rarey, M. (2010) Drawing the PDB: Protein-Ligand Complexes in Two Dimensions. ACS Med. Chem. Lett. *1*, 540-5.

Zalkin, H., and Smith, J.L. (1998). Enzymes utilizing glutamine as an amide donor. Adv. Enzymol. Relat. Areas Mol. Biol. *72*, 87–144.
